# Beyond Extracellular Vesicle (EV) Hype: Practical Solutions and Remaining Hurdles in EV Research, Manufacturing, and Clinical Translation

**DOI:** 10.1002/advs.202521913

**Published:** 2026-04-07

**Authors:** David J. Lundy, Zoe L. Chau, Sheng‐You Chen, Nanami Fujisawa, John J. Hill, Barnett M. Hsu, Joanne K. Liu, Karol W. Mai, James J. Lai

**Affiliations:** ^1^ Department of Materials Science and Engineering National Taiwan University of Science and Technology Taipei Taiwan; ^2^ Department of Bioengineering University of Washington Seattle Washington USA; ^3^ Graduate Institute of Biomedical Materials and Tissue Engineering Taipei Medical University New Taipei City Taiwan; ^4^ International PhD Program in Biomedical Engineering Taipei Medical University New Taipei City Taiwan; ^5^ David Geffen School of Medicine University of California Los Angeles California USA; ^6^ Woodruff School of Mechanical Engineering Georgia Institute of Technology Atlanta Georgia USA; ^7^ Graduate School of Pure and Applied Sciences University of Tsukuba Tsukuba Japan; ^8^ Department of Medicinal Chemistry University of Washington Seattle Washington USA; ^9^ Department of Medicine Stanford University Medical Center Palo Alto California USA

**Keywords:** biomanufacturing, extracellular vesicles (EVs), quality attributes, regulatory framework, therapeutic translation

## Abstract

Extracellular vesicles (EVs) are nanoscale mediators of intercellular communication with diverse molecular cargoes that reflect their cell of origin. Advances in isolation, detection, and single‐particle analytics have revealed increasing molecular and functional heterogeneity, while exposing limitations in how EV identity and activity are currently defined. The field has expanded rapidly; however, translational progress is constrained by an incomplete mechanistic understanding, a lack of standardized measurements, and inconsistencies in regulatory classification. This review provides a critical synthesis of current EV research from an analytical and translational perspective, emphasizing the measurement science needed to define, compare, and benchmark EV preparations as therapeutic products. We discuss evolving regulatory frameworks and recent updates to the MISEV guidelines, highlighting the need for operational definitions grounded in source material, isolation method, and molecular markers. Updated workflow considerations are presented across EV production and characterization, with a focus on orthogonal analyses, quantification of co‐isolates, and potency assays that support reproducibility and quality control. Together, these advances and ongoing challenges underscore the need for analytical rigor to transform EV research from descriptive studies into a reproducible and quantitative measurement science.

## Graphical abstract

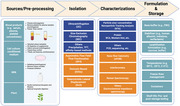

## 1 Introduction

### 1.1 Extracellular Vesicles in the Secretome: Updated Definitions

Over recent decades, we have come to more fully appreciate the role of the cell secretome, which comprises cytokines, lipoproteins, protein complexes, hormones, metabolites, and extracellular particles (EPs) [1, 2]. Together, these shape tissue homeostasis, growth, pathology, repair, and aging. Among these factors, extracellular vesicles (EVs) have captured substantial attention as putative biological nanocarriers capable of delivering cargo to both local and distal sites. EVs are defined as non‐proliferating, lipid‐bilayer bound particles released by living cells, comprising a larger population of EPs that includes non‐vesicular EP (NVEP) protein–RNA complexes and lipoproteins [3]. Consensus guidance (Minimal Information for Studies of Extracellular Vesicles, MISEV23) recommends that studies should rely on operational definitions grounded in the source material and isolation methods, combined with the resulting particle concentration, molecular markers, and functions. MISEV23 encourages the use of the term “EV” as a broad baseline term, and discourages the use of labels such as exosome or microvesicle without supporting size or biogenesis data [3].

Several recent reviews, including our own, have summarized EV biology, isolation and characterization methods, engineering strategies, and therapeutic applications across multiple disease settings [4]. This article aims to complement that literature by focusing less on cataloging reported benefits and more on practical translational issues that affect all indications. That includes how EV preparations are defined, measured, manufactured, benchmarked, and regulated. Using this framework, we consider the “EV preparation”, encompassing production, isolation, and characterization, rather than an idealized “pure EV” fraction, as the relevant unit for therapeutic development. Accordingly, we primarily discuss EVs derived from cell culture and blood products, and to a lesser extent, milk EVs and plant‐derived nanovesicles, with emphasis on reproducibility, critical quality attributes, and clinical translation.

### 1.2 Pre‐Clinical Promise, and the Extract/Drug Dichotomy

EVs have attracted attention as both therapeutics and biomarkers, and this review focuses primarily on their therapeutic applications. Here, natural/native EVs can be collected and purified from solutions, such as cell culture media, blood, or milk, and then administered without further modification [5, 6, 7, 8]. Alternatively, EVs can be engineered to carry varying amounts of specific cargo, or loaded with exogenous compounds and used as delivery vehicles for other therapeutics. Thousands of studies report EV efficacy in pre‐clinical disease models spanning myocardial infarction (MI) and wound healing to neurodegeneration, infectious diseases, and cancer [9, 10, 11, 12]. Across these models, EVs have been delivered across various routes, including inhalation, intranasal, intravenous, subcutaneous, topical, ophthalmic, and others, generally with low toxicity and positive outcomes. As of late 2024, more than 470 clinical trials were registered across >200 indications [13], and a growing number of private clinics, dermatologists, and cosmetic manufacturers now offer “exosome” products and interventions directly to consumers. Consequently, EVs are simultaneously viewed through two lenses: first, as regulated biologics with defined source materials, controlled manufacturing, measurable critical quality attributes (CQAs), and potency assays, and second, as undefined natural extracts deployed across diverse settings. Here, we evaluate EVs primarily guided by the standards applied to biologics. Despite rapid progress, fundamental questions about EV biology, mechanisms, and study reproducibility remain, posing hurdles to their translation. These challenges, together with persistent hype, evolving regulatory frameworks, and manufacturing challenges, frame the key issues we examine in the following section.

## 2 The Road to Translation: Scientific, Regulatory, and Technical Hurdles

In the sections that follow, we critically examine where current evidence supports claims of therapeutic efficacy, and where prevailing assumptions about purity, targeting, delivery, and mechanism remain less certain.

### 2.1 Hype and Knowledge Gaps

#### 2.1.1 The “Pure EV” Paradigm

A common framing in published EV studies is: “EVs from a certain source solution carry specific miRNA/protein molecules that can target specific tissues/cells to produce a therapeutic effect” [14, 15, 16, 17]. While strong evidence may be presented, the framing is inherently reductionist since, in practice each EV preparation (regardless of source or isolation method) contains hundreds of RNAs and proteins, plus lipids, metabolites, and non‐vesicular co‐isolates that are rarely quantified; “EV preparation” is therefore a more accurate term. With current analytical technologies and an incomplete biological understanding, “pure” EVs cannot yet be isolated nor consistently defined [3]. Inherent variability is also a major challenge—EVs are heterogeneous and dynamic, with markers, cargo, and function varying between, and within, cell types [18, 19, 20, 21, 22]. Even under tightly controlled in vitro conditions with consistent medium composition, culture surface, oxygenation, confluence, and harvest timing, a single cell population secretes multiple EV subpopulations with distinct cargo and functions [23]. As such, the employed isolation methods further affect the composition and function of the final EV preparation, by enriching or reducing certain subpopulations [24, 25]. In addition, EVs can acquire a protein corona (e.g., apolipoproteins, albumin, complement) that co‐purifies and may contribute to observed activity [26]. Therefore, it is very challenging to define, quantify, and enrich the truly active component(s) of an EV preparation, establish CQAs, and distinguish functional cofactors from true contaminants. As one example of seldom‐measured cargo constituents, cell‐secreted EVs have recently been found to contain physiological (copper, zinc, magnesium) and non‐physiological (lead, aluminum) metals, which can be transferred to recipient cells and participate in redox reactions, or contribute to toxicity [27]. To address this, the most recent ISEV recommendations urge researchers to report EV preparations in detail, including the source, isolation stack, quantitative metrics (such as size distribution and particle‐to‐protein ratio), and ideally the quantification of co‐isolates [3]. Datasets may be deposited with repositories and annotated with EV‐TRACK to help improve reproducibility in the field. Newer analytical technologies, including single‐EV approaches, may shed light on heterogeneity, subpopulations, and marker stoichiometry within EV preparations [3, 28]. These will be discussed in Section 4.1.

#### 2.1.2 Cargo–Function Ambiguity Limits Mechanistic Understanding

Definitively linking specific cargo in natural EVs to a particular therapeutic function or outcome is challenging. To highlight one example, miR‐21 is frequently found in EV preparations and has been reported to play roles in both pathophysiology and therapy of MI [29, 30, 31, 32, 33, 34, 35, 36]. Specifically, miR‐21 associated with mesenchymal stem/stromal cell (MSC)‐derived EVs has been reported to possess multiple beneficial activities, including reducing cardiomyocyte apoptosis via PDCD4, PTEN, Peli1, and FasL, protecting post‐MI hearts through macrophage KBTBD7 targeting and dampening inflammation, and improving contractility and calcium handling in hypoxic cardiomyocytes through PI3K signaling [29, 30, 31]. On the other hand, studies have shown that miR‐21 can promote fibroblast activation and fibrosis via Smad7 targeting following MI [32]. Interventional studies have also shown seemingly opposing results: delivery of miR‐21 inhibitors using blood‐derived EVs improved survival and cardiac function after MI [33], whereas non‐EV nanocarriers (liposomes and mesoporous silica nanoparticles) delivering miR‐21 mimics reported functional benefits in rat and pig models [34, 35]. Thus, when abundant miR‐21 is detected in an EV preparation intended for MI therapy, should it be considered an important, active therapeutic component, or as an unwanted, harmful contaminant? It is likely that proportions of other miRNAs, in addition to proteins, lipids, metabolites, and co‐isolates in the EV preparation, also contributed to the overall outcomes. These examples were intended to highlight that even a single abundant cargo constituent, within a single disease model, can have context‐dependent effects that differ based on the EV source, experimental model, and other factors such as the other constituents of the EV preparation.

This complexity clearly frustrates efforts to define clear active‐ingredient profiles or straightforward potency metrics for EV therapeutics. Consistent with this, studies have shown that differences in bulk proteomic or miRNA cargo did not reflect functional immunomodulatory activities of EVs [26]. Thus, widely‐used EV characterization methods such as proteomics, miRNA profiling, and physicochemical analyses are generally unable to reliably predict the function of EV preparations based soley on measuring their cargo. To address these points, researchers are encouraged to use gain‐ and loss‐of‐function experiments, to use cargo‐null recipient models to quantify EV net delivery, and to explore wider dose‐response ranges [3]. If the ‘active’ components of an EV preparation cannot be unambiguously localized to a vesicle lumen or membrane, then we believe claiming EV mechanisms at the single‐cargo level is premature. The next question, then, is how EVs deliver those cargos and enact their therapeutic effects.

#### 2.1.3 Cellular Uptake, Intracellular Delivery, and Effects

Another limitation in our biological understanding concerns how EVs distribute in vivo and engage target cells, such as internalization and cargo delivery [37]. The traditional concept is that EVs enter cells, discharge cargo, and thus trigger downstream effects. Supporting this, many reports have shown enhanced delivery of exogenous substances, such as small molecules, RNAs, peptides, and polymers, when encapsulated in EVs [7, 37, 38]. In one study, natural EVs were directly compared with synthetic lipid nanoparticles using a highly sensitive CRISPR/Cas9‐based RNA transfer reporter system [39]. Here, EVs prepared from cultured MDA‐MB‐231 and A321 cells appeared to have far higher efficiency in terms of RNA delivery into HEK293 cells [39, 40]. However, even under favorable in vitro conditions where substantial EV uptake (∼30% over 24 h) was documented, only a small fraction of recipient cells expressed the reporter system. This shows that EV entry into a cell does not guarantee cytosolic delivery of EV cargo, endosomal escape, or subsequent gene regulation [39]. Further complications arise from technological limitations of measuring EV uptake, and many early studies in the field inferred delivery by labeling EVs with lipophilic dyes such as DiI [41]. Newer studies with improved EV tracking methods (such as nanoluciferase and reporter proteins) have revealed a more complex picture of EV‐cell interactions [37]. For example, using luciferase or fluorescent‐tagged cargo proteins to track HeLa cell EV uptake by HeLa cells, only ∼1% of the input EVs (1 µg/ml) were internalized after 1 h and, of those, only ∼20%–30% released soluble cargo into the cell cytosol. Interestingly, EV uptake was temperature‐dependent, and cargo release required endosomal acidification and was blocked by IFITM1/3, indicating a fusion‐like, pH‐dependent step downstream of EV entry. This indicates that internalization was driven primarily by endocytosis, with little contribution from specific membrane protein binding in this experimental system [42]. A separate study used a dual‐reporter system in which HEK293T cells expressing NanoLuc‐fused constructs targeted to specific organelles (CoxVIII‐mitochondria, H2B‐nucleus, and Lyn‐membrane) were used to produce EVs that could be both quantified (luminescence) and localized (fluorescence) after uptake. After isolation of CD9^+^/CD63^+^ HEK293‐EVs by size‐exclusion chromatography, uptake into recipient (HeLa) cells was quantified by luminescence, and delivery was also determined to be <1 %. The study also showed that recipient cells re‐released intact, CD9^+^ Nluc‐labelled EVs after uptake, effectively reducing net delivery [43]. Thus, in these well‐controlled studies using highly‐specific tracking methods, internalization of EVs did not inherently result in successful cargo delivery or intracellular activity. However, it should be noted that all uptake/internalization studies are subject to the effects of the dose (particles or protein per cell), which in turn, depends on the purity of the EV preparation. Additionally, it is possible that alternative EV sources and different recipient cells may exhibit varying levels of complementarity and specific uptake. Lastly, some studies have shown that EV internalization may not even be required for overall therapeutic activities, such as those mediated by EVs binding to cell‐surface receptors [44]. EVs have also been shown to function as decoy receptors for soluble mediators via their surface receptors [45, 46]. For example, CCR2^+^ MSC‐EVs may deplete pro‐inflammatory extracellular CCL2 in kidney injury, and macrophage EVs with transferrin receptors can absorb transferrin‐bound iron, thereby reducing ferroptosis after MI [45, 46].

Taken together, EV internalization and uptake remain relatively incompletely understood, do not always equate to functional cargo delivery, and may not be necessary for some therapeutic effects. Emerging genetic and bioluminescent reporters (e.g., luminal luciferase constructs) can help distinguish binding/uptake from productive cargo release in real time [47].

#### 2.1.4 Biodistribution, Biological Barriers, and the ‘Targeting’ Mirage

Two recurring claims are that EVs (i) can readily cross biological barriers, such as the blood–brain barrier (BBB), and (ii) possess innate targeting towards diseased tissues. However, findings vary across studies depending on the EV sources, disease models, labeling methods, and uptake measurements. Regarding BBB permeability, numerous preclinical studies in central nervous system (CNS) disease models, using EVs to treat brain tumors, neurodegeneration, and traumatic injury, report functional benefits such as improved survival, cognition, and behavior [12]. However, much of the purported BBB passage was inferred using lipophilic dye‐labelled EVs and whole‐organ imaging (e.g., IVIS), which report bulk accumulation in the tissue rather than cell‐specific internalization, cargo release, or function [41]. A comparative study of EV labeling methods concluded that covalent attachment of radiolabels to the EV surface was optimal for quantitative biodistribution studies. Upon using this method, it was found that brain entry of Expi293F‐EVs in healthy mice was negligible [48]. In non‐human primates, a dual‐reporter system also showed negligible uptake of Expi293F‐EVs in the brain after intravenous administration, and even lower uptake after intranasal administration, which was expected to increase brain delivery [49]. Barrier disruption (due to injury/inflammation) may increase deposition, and it is possible that EV preparations from other sources may exhibit superior brain delivery [50], but these newer studies challenge previous blanket claims of efficient BBB traversal by EVs. At the same time, these findings do not preclude beneficial activity in brain disorders, but peripheral mechanisms such as immunomodulation may also contribute to therapeutic effects.

EV “targeting” to solid peripheral tumors faces similar challenges. Many studies have shown anticancer activities using natural EVs or EVs loaded with therapeutic compounds [48, 51, 52]. However, when covalently radiolabeled EVs were injected into tumor‐bearing mice, total tumor accumulation remained below ∼2% of the injected dose per gram (%ID/g), with uptake into tumor cells likely being significantly lower [48, 53]. Across routes and indications, a systematic review of in vivo EV biodistribution showed that liver, spleen, lung, and kidney are the dominant sites of deposition, with limited accumulation elsewhere, including tumors, where reported accumulation is extremely variable between studies [41]. A recent study using SPECT to track Technetium‐99m radiolabelled EVs from umbilical cord MSCs in mice showed a rapid distribution phase of <1 min and an elimination phase of ∼25 min after intravenous administration [54]. The source and composition of EV preparations appear to be important for distribution and targeting, and likely explain some of the variability observed in the literature. For example, Liam‐Or and colleagues found that an albumin‐rich surface corona significantly changed MSC‐EV targeting, reducing uptake by hepatic macrophage cells and enhancing uptake by hepatocytes and endothelial cells [55]. Similarly, Rosenkrans and colleagues showed that EVs from MSCs, macrophages, or melanoma cells showed different biodistribution profiles in immunocompetent, immunodeficient, and tumor‐bearing mice [56]. In their study, EVs were radiolabelled and tracked by PET, resulting in a calculated circulatory half‐life of 12‐14 h, which is significantly longer than previously‐described studies. This again illustrates that the labeling method and measurement method, as well as EV source and EV purity, can significantly impact their calculated biodistribution. Recently, Cho and colleagues developed a label‐free method using human mitochondrial DNA quantitative PCR to track Expi293F EV mimetics in rodents, therEby circumventing potential artifacts from exogenous dyes or reporter proteins. Fluorescence signal from co‐labeled EVs peaked at 30 min post‐intravenous administration, with the liver showing the highest and most sustained accumulation across timepoints (0.5, 3, and 24 h), further corroborating rapid hepatic clearance observed with other tracking modalities [57]. These findings of rapid clearance and low uptake by target tissues do not preclude biological effects, but many previous statements of “homing” or “precision targeting” by EVs tend to describe modest enrichments in uptake over low baselines. Interestingly, a very recent study used in vitro and in vivo models to challenge the commonly stated idea that EVs naturally act over long distances. Colombo and colleagues showed that the majority of released EVs were internalized by adjacent cells, with an estimated 80% of the signal retained within 40 µm of the originating cell in a tumor microenvironment [58].

Lastly, EVs have been widely reported to have a high degree of immunocompatibility. A recent study measured delivery of full‐length VEGF‐A mRNA to ischemic injury sites by fibroblast‐derived EVs, and found fewer innate or adaptive immune responses than when mRNA was delivered by viral vectors or lipid nanoparticles [7]. However, other studies have shown that repeated dosing of EVs can still elicit humoral responses resulting in accelerated circulatory clearance [59, 60]. The topic of EV immunogenicity has been recently reviewed in detail elsewhere [60].

Taken together, it appears that certain EV populations may possess properties more favorable for specific indications, but absolute statements that EVs readily traverse biological barriers, intrinsically target diseased cells, or evade the immune system are not well supported by the evidence. The observed variability is likely due to both biological and methodological variability, including the source and purity of the EV populations, and the labeling methods, which may also affect biodistribution. As the field advances, more precise methods for characterizing and tracking EVs should significantly enhance our understanding of EV biodistribution and uptake.

### 2.2 Commercial, Clinical, and Regulatory Development of EV/Secretome Therapeutics

Commercialization serves as the culmination of translational development, enabling EV technologies to be realized as therapeutic interventions. In the following sections, we briefly highlight some leading translational efforts and regulatory approaches, while illustrating some apparent conflicts between clinical ambition and scientific foundations. EVs currently inhabit two parallel worlds: (i) the regulated biologic‐drug pathway pursued via IND (Investigational New Drug)/IMPD (Investigational Medicinal Product Dossier)‐backed clinical programs, and (ii) a diffuse market of cosmetic/wellness offerings that promote “exosomes” for indications as diverse as aging, autism, diabetes, osteoarthritis, and allergies.

#### 2.2.1 Leading Clinical Translation Efforts

For clinical translation, there is significant momentum driving EV‐based biologics into randomized controlled clinical trials, although they are still mostly at preliminary stages [13]. Here, we highlight some leading examples. The first widely publicized EV‐based therapeutic to reach clinical testing was ExoFlo, a BM‐MSC‐derived EV product [61]. An initial open‐label study in patients with severe COVID‐19–associated ARDS (*n* = 24) demonstrated feasibility and safety, though its non‐randomized design limited interpretation of efficacy. Subsequent randomized, placebo‐controlled trials [62] confirmed safety and suggested a mortality benefit in a patient subgroup, supporting continued investigation. While the studies drew attention due to a lack of transparent reporting of dose, purity, and CQAs [63], these trials nonetheless represent important milestones toward clinical translation. A multicenter Phase 3 trial (EXTINGUISH‐ARDS, NCT05354141) is now underway, with results expected in 2027, reflecting consistent momentum in EV‐based therapeutics.

Another leading example of EV therapeutic translation is RION, a Mayo Clinic spin‐out. RION has developed Purified Exosome Product (PEP), a shelf‐stable lyophilized platelet‐derived EV product with a reported room‐temperature shelf life of 24 months. PEP has been evaluated across multiple indications, with diabetic foot ulcers (DFU) representing the most advanced program. A Phase 2 multicenter trial (*n *= 59) evaluating topical PEP for DFU (NCT06319287) has completed enrollment, and in January 2026, INTENT Biologics (a RION subsidiary) received FDA Fast Track designation for PEP Biologic in DFU, with a pivotal Phase 3 trial planned for 2026 [64]. Additional IND‐enabled studies are underway for knee osteoarthritis (Phase 1b) and intradermal aesthetics applications (Phase 1, NCT06429033). In September 2025, RION announced a cGMP manufacturing partnership with Lonza for commercial‐scale production of PEP [65].

Another allogeneic platelet‐derived EV, Plexaris (ExoPharm Limited) is prepared using a technology termed ligand‐based exosome affinity purification (LEAP), which is based on ion‐exchange chromatography [10]. In a first‐in‐human clinical trial (Plexoval II study, ACTRN12620000944932), healthy volunteers received bilateral punch biopsies, and Plexaris was administered subcutaneously surrounding the injury site on one arm. The study used a blinded, placebo‐controlled design, and Plexaris was found to be safe and well‐tolerated. The company has since wound down naïve EV product development, but the LEAP technology remains available for licensing.

#### 2.2.2 Brief Overview and Updates of Regulatory Frameworks

Regulatory guidelines are important not only for ensuring the safety and efficacy of EV products but also for streamlining pre‐clinical and clinical studies. If EVs are to be classified and used as serious biological medicines, guidelines must compel the industry to establish clarity around dosing regimens, the assessment of material potency, and the determination of product purity. However, these are the very issues that are still handled inconsistently at the basic research level, and hype and demand for EV therapies have outpaced regulatory enforcement. The regulations governing EV research and commercialization vary between countries, as reviewed by others [66, 67, 68]. ISEV has also established the Regulatory Affairs and Clinical Use of EV‐based Therapeutics Task Force (www.isev.org/regulatory‐affairs‐task‐force), which aims to identify regulatory guidances relevant to EVs as investigational new drugs.

Regulatory oversight of EV products varies widely across jurisdictions, reflecting differences in how these materials are classified relative to biologics, gene therapies, or regenerative products. In the United States, the FDA's stance is clear—‘exosome’ products intended for therapeutic use are regulated as drugs or biologics requiring premarket approval, and none are currently authorized for sale [69]. Similarly, the use of human‐derived materials in cosmetic products is explicitly prohibited in the European Union (EU) and the United Kingdom (UK) under EU/UK 1223/2009 [70, 71]. In the EU, the Committee for Advanced Therapies (CAT) classifies EV‐based products on a case‐by‐case basis, generally as biological medicinal products, except those that deliver recombinant nucleic acids, which fall under the category of gene therapy medicinal products [72]. In a recent (January 2026) example, the European Medicines Agency (EMA) CAT placed a miR‐140‐loaded Wharton‐Jelly hTERT MSC‐derived EV product within the committee's advanced therapy medicinal products (ATMP) classification workstream [73]. Japan's Act on the Safety of Regenerative Medicine does not currently cover EVs, allowing limited clinical use under less oversight; the Pharmaceuticals and Medical Devices Agency has since issued new guidance to address this gap [74, 75, 76, 77]. South Korea and Taiwan have taken more proactive steps, issuing EV‐specific guidelines in 2018 and 2025, respectively, outlining standards for manufacturing, quality control, and clinical assessment [67, 78].

#### 2.2.3 Widespread Availability of Unapproved Products

In the USA, EV therapies fall under the FD&C Act and PHS Act §351 as drugs/biologics, but some companies have attempted to frame secretome, “exosome” or amniotic products as §361 HCT/Ps (Human Cells, Tissues, and Cellular and Tissue‐Based Products) to bypass premarket review. However, the FDA has issued multiple public notices clearly stating that EV interventions for treating diseases or conditions do require premarket authorization [79, 80]. Despite this consistent messaging, the persistent marketing of products has prompted the FDA to issue public alerts and send warning letters to several companies [69, 81, 82, 83]. For example, in September 2025, the FDA issued a warning letter to New Life Medical Services, LLC, concluding that its umbilical cord–derived (Restor+, Regain, Renyte), amniotic fluid–derived (ReCyte/Cytosomes), and exosome/“extracellular vesicle” product (Rexo) are unapproved new drugs and unlicensed biological products, and that the HCT/Ps fail the minimal‐manipulation and homologous‐use criteria under 21 CFR 1271.10(a). Similarly, in January 2025, the US FDA warned Chara Biologics for marketing ‘CharaExo’, containing ‘amniotic fluid‐derived exosomes’ for infertility, osteoporosis, diabetes, autoimmune diseases, and many other disorders [83]. In both cases, the warning letters cited current good manufacturing practice (cGMP) violations, misbranding, and failing to obtain a valid biological license application (BLA) under section 351(a)(1) of the PHS Act, 42 U.S.C. § 262(a)(1). The letters state, “*such licenses are issued only after showing that the product is safe, pure, and potent*”, indicating that the US FDA is approaching EVs based on a biologics framework. In December 2024, the FDA warned INCELL Corporation (contract manufacturer) and XO Biologix (marketer of MaviX) that their amniotic fluid–derived product is an unapproved new drug and an unlicensed biological product [84]. Here, they explicitly noted that secreted bodily fluids like amniotic fluid are generally not HCT/Ps under 21 CFR 1271.3(d). XO Biologix's materials described MaviX as a “lyophilized, sterile product composed of enriched lipoprotein vesicles,” while the company's website described “repair and regeneration” benefits. However, in a post‐letter interview, INCELL's CEO/CSO disagreed that the product is a drug, stating, “INCELL is not a drug manufacturer” [85].

Despite regulatory agencies from multiple countries urging caution, some influencers, clinicians, and companies have contributed to public demand for these products by creating hype around purported benefits. Many cosmetic products containing ‘exosomes’ are available, and some clinics have also begun offering secretome or “exosome”‐based interventions for skin care, hair loss prevention, and anti‐aging [86, 87]. However, the source, quality, and dose of the product are often opaque. While it is certainly plausible that EVs may be beneficial for these indications, claims of rejuvenation are not supported by clinical trial data, and these interventions carry risks. Both scientific case reports and popular media outlets have described serious adverse events following cosmetic ‘exosome’ interventions, including the formation of papules, granulomas, and tissue necrosis [86, 87, 88, 89]. The US FDA has also described multiple serious adverse events in patients who received unlicensed exosome products [90]. Thus, it is clear that production and/or analytical standards for these products were inadequate.

### 2.3 Technical Challenges in Manufacturing, Scaleup, and Translation

The clinical translation of EVs is currently hindered by a chain of technical hurdles, including EV heterogeneity, isolation methods, and analytical/characterization methods. Here, we summarize the most pressing technical issues currently limiting the translation of EV therapeutics.

#### 2.3.1 Challenges With Heterogeneity and Standardization

EV preparations vary greatly between sources, within the same source (subpopulations), and across different collection, isolation, and characterization methods, posing challenges for standardization. These variables are well documented in many recent studies and International Society for Extracellular Vesicles (ISEV) guidelines [18, 19, 23, 25, 91, 92]. For example, the aforementioned studies (Section 2.1.2) reporting divergent roles of miR‐21 for MI used EVs collected from various sources and employed different isolation/concentration protocols [29, 30, 31, 32, 36]. Thus, while the studies all focused on EV miR‐21, the different study outcomes were likely significantly influenced by the heterogeneity associated with EV preparations.

As an example of heterogeneity, it has been shown that the secretome from a single cell type contains a variety of EVs, which can be divided into subpopulations exhibiting different cargo and activities. For example, EVs secreted from cardiac progenitor cells (CPC‐EVs) were separated by size‐exclusion chromatography (SEC) and divided into three subpopulations [23]. Each had different proteomic cargo, surface markers, and degrees of uptake by cardiomyocytes, fibroblasts, endothelial cells, and macrophages. Thus, differing pro‐angiogenic and anti‐fibrotic potencies of EV preparations were measured at the same given dose, despite originating from identical source materials. This illustrates the importance of standardizing and fully reporting EV isolation methods. Regarding dosing, both protein‐ and particle‐based doses depend on the quality of the EV preparation, and neither approach fully accounts for co‐isolates. Dosing by the amount of a particular peptide or nucleic acid is another potential approach; however, as mentioned earlier, each EV preparation contains hundreds of biologically active substances, and it is unclear which of the components, or combination of components, are the ‘active’ therapeutics. Since EV cargo is also unequally distributed throughout a preparation, profiling the content of a bulk EV preparation may not adequately predict functional activity [26]. Thus, EV heterogeneity is a key challenge to ensuring batch‐to‐batch consistency and predictable therapeutic effects. Single‐particle analytic technologies (which will be described later, in Section 4.1) may be able to better standardize batches and establish CQAs in the future.

#### 2.3.2 Challenges With Production Scaling

Scaling EV manufacture from bench‐scale, research‐grade materials to larger‐scale, good manufacturing practice (GMP), clinical‐grade materials, often referred to as the “scale‐down modeling” in process development circles, is a second major hurdle. Scaling must be considered for both upstream (source, cell culture) and downstream (isolation method, purification, concentration) methods [93]. At the upstream level, cell‐derived EVs will require the mass production of GMP‐grade cell‐conditioned reagents and materials for use in GMP manufacturing processes. The sources of these materials will likely be different from those used in supplying a typical research laboratory. For example, EVs obtained from research‐grade cultures using static two‐dimensional plasticware may not analytically or functionally represent the EV preparations obtained from GMP‐grade cell culture used for small‐scale production runs. In turn, these may not represent the higher‐output, larger‐scale perfusion bioreactors and three‐dimensional culture systems used in process development manufacturing suites [20, 94]. Illustrating this, Jeske and colleagues compared human MSC‐EV production between a conventional 2D culture system (Corning CellBIND T‐flasks) and 3D microcarriers in bioreactors (0.1 and 0.5 L) to examine the effects of production scaling [95]. After 5 days, cell expansion in 2D culture was >50% higher than in the bioreactors, and 2D cultures exhibited higher glucose and lower lactate concentrations, likely reflecting their lower initial seeding density. However, when normalized to cell number, bioreactor cultures exhibited significantly higher glucose consumption and lactate production, although the lactate‐to‐glucose molar ratio remained consistent (1.5–1.8) across all conditions. Importantly, EV secretion normalized to cell number was nearly threefold higher in bioreactors compared with 2D culture. Bioreactor‐derived EVs also showed stronger expression of HSC70 and CD81 (western blot) and upregulation of multiple EV miRNAs (miR‐10, 19a, 19b, 21, 30b, and 132) relative to 2D‐derived EVs. Thus, scaling up EV production altered the yield and cargo of the resulting EV preparations. Other studies have reported similar findings, with 3D/microcarrier culture, bioreactors, hypoxia, and dynamic cultures increasing the yield of EVs two to five‐fold compared to static 2D cultures [96, 97, 98].

Similarly, many basic research publications use EV‐depleted FBS (either commercial or prepared in‐house) to promote cell proliferation, but “EV‐depleted” FBS leaves behind substantial numbers of bovine vesicles and small RNAs that co‐isolate with cellular EVs and are not suitable for clinical applications [99, 100]. Human platelet lysate (HPL) is a xeno‐free alternative, but it introduces P‐EVs and growth factors (e.g., CD41/CD61‐positive vesicles; PDGF, TGF‐β, and platelet‐derived miRNAs) that can change both yield and apparent potency of the resulting EVs unless defined xeno‐free media are used for production runs [101]. This difference in medium composition again alters cell behavior and affects the composition and function of EV preparations [100]. Together, these findings highlight that EV yield and cargo composition are strongly influenced by the materials and media used in scaled‐up production platforms [95]. In terms of basic research, there are now commercially available kits (e.g., RoosterBio) for 3D perfusion bioreactor culture of MSC at scales up to 50 litres, with integrated EV harvesting. This will help to reduce the gap between basic EV research and the EV production methods required for clinical translation.

Lastly, downstream isolation methods can also pose challenges when research‐grade processes are difficult to scale up for larger‐scale manufacturing processes. For example, differential/gradient ultracentrifugation (UC) is commonly used in EV research to isolate EVs from cell‐conditioned culture medium [3]. However, these are low‐throughput methods (limited by maximum volume). The resulting operations require frequent operator interventions, including during the batch steps such as balancing tubes, resuspending, and washing pellets, all of which increase the risk of contamination. UC can also aggregate EVs and co‐isolate NVEPs, thereby affecting the purity of the preparation and introducing variability [102]. SEC is also commonly used in research because it is gentler, more effective at removing small proteins, and can be scaled by enlarging the column volume. However, SEC dilutes EV preparations and therefore typically requires downstream concentration of the collected fractions [103, 104]. The concentration steps (such as centrifugation or filtration) again require manual intervention and introduce variability in the final EV preparation. Thus, many processes commonly used for basic EV research do not scale well to clinical‐scale production, creating a hurdle for translation to GMP manufacturing processes for large‐scale supplies of clinical‐grade EV products.

#### 2.3.3 Challenges due to Limitations in Analytical Technologies

Once EVs have been enriched from their original source, they must be characterized. Using the US FDA framework of safety, purity, and potency, this means that the composition and activity of the preparation should be known [105]. However, as described earlier, linking cargo to potency is an area where the EV research field has yet to reach conclusive answers. In terms of safety, blood products are an example of a naturally derived biological products with a strong clinical track record [106]. Safety testing and CQAs are well established and utilized worldwide. Thus, EV preparations derived from transfusion‐ready blood products such as plasma or platelet concentrates are strong candidates for translation, as these products already contain EVs. Similarly, MSC‐EVs are also highly feasible, as live MSCs (which secrete MSC‐EVs after transplantation) have been used in clinical trials [107]. Here, cell identity/quality is anchored to well‐defined surface panels, viability, and differentiation assays, while dosing is unambiguously based on the number of live cells.

By contrast, EV preparations are more challenging to characterize than cells or blood products due to our more limited vesicular analytical capabilities. Commonly employed EV analytical methods, such as nanoparticle tracking analysis (NTA), dynamic light scattering (DLS), tunable resistive pulse sensing (TRPS), western blot, proteomics, and quantitative polymerase chain reaction (qPCR), produce population‐based metrics [103] that can mask subpopulations, co‐isolates, and heterogeneity. Thus, dosing based solely on total particle counts or protein amount does not guarantee that consistent preparations will be administered. In terms of safety, some straightforward assays can be performed, including endotoxin screens, sterility tests, and quantification of specific proteins/nucleic acids [93]. However, establishing generic CQA parameters for EV products will be extremely challenging, and these are more likely to need to be determined on a case‐by‐case, application/indication‐specific basis, as discussed in Section 4.2 [93].

## 3 EV Processing

**SCHEME 1:**
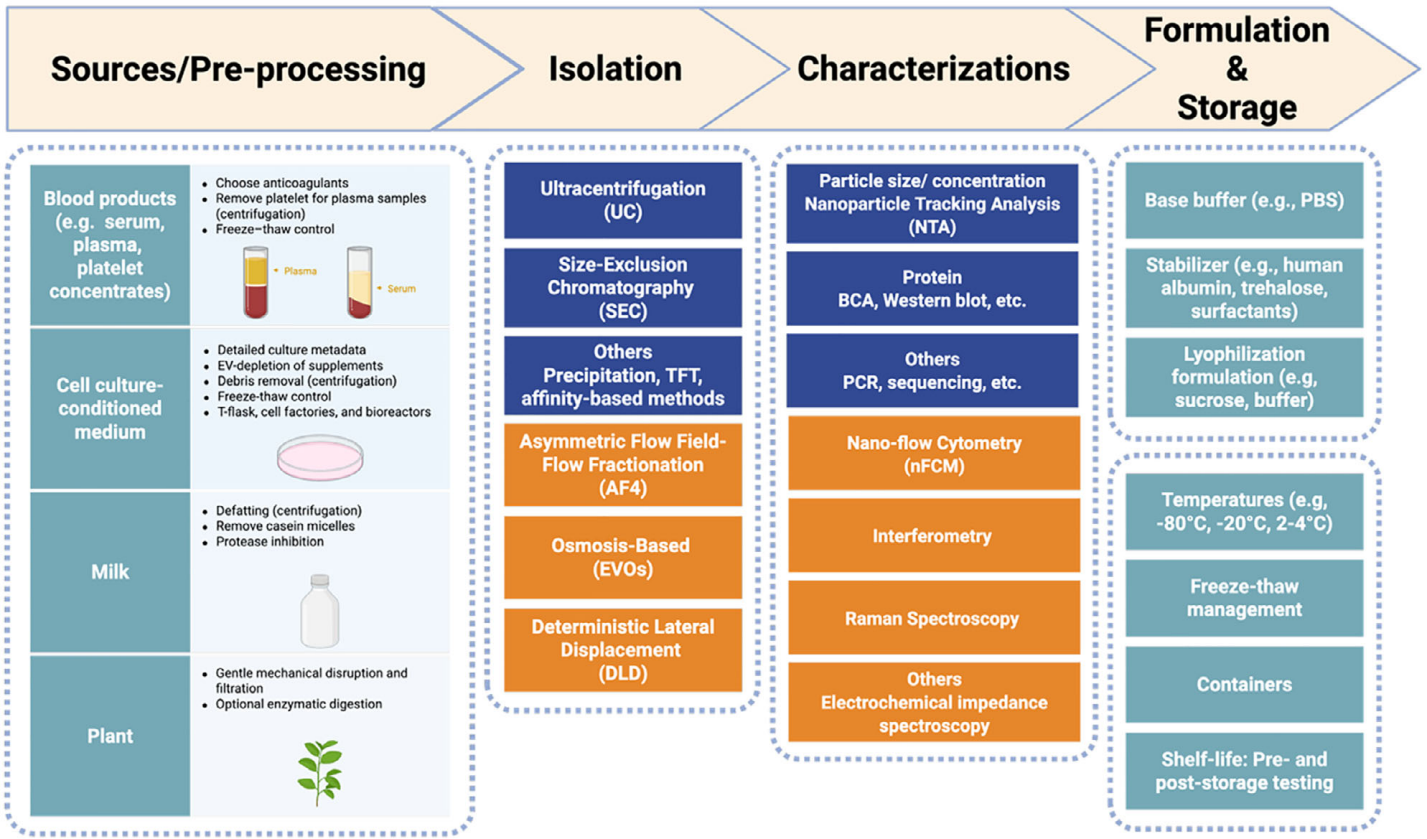
A brief EV workflow. For Isolation and Characterization, blue color indicates established approaches and orange color indicates novel methods included in this review. Created in BioRender. Lai, J. (2026) https://BioRender.com/569yrs.

All EV workflows, as outlined in Scheme 1, begin with source collection followed by pre‐processing or cleanup. Pre‐processing often includes centrifugation to remove platelets, cell debris, or fat droplets from blood, cell culture medium, and milk, respectively [108, 109, 110]. For plant‐derived materials, gentle mechanical disruption, often accompanied by optional enzymatic digestion, is typically applied [111, 112]. Following pre‐processing, the clarified solution is subjected to EV isolation—normally a multistep sequence of unit operations—and subsequently characterized to establish its identity, purity, and potency. Finally, the resulting EV preparation is formulated and stored under defined conditions to preserve stability and function prior to downstream use or clinical administration. Formulation typically involves buffer exchange and concentration adjustment (and, where appropriate, excipient selection and sterile handling). At the same time storage parameters such as container type, temperature, freeze–thaw exposure, and storage duration should be treated as critical variables. Re‐testing after storage using stability‐indicating physicochemical, compositional, and potency assays helps confirm lot integrity and supports comparability across batches and studies. The following sections first summarize selected reported therapeutic activities by source and then discuss the source‐specific processing constraints that influence real‐world manufacturing.

### 3.1 Therapeutic Effects of EVs from Different Sources and Their Processing Challenges

As discussed in Section 2.2, the two sources currently showing strong translational momentum are MSC‐EVs and P‐EVs [13, 105, 106, 113, 114, 115, 116, 117, 118, 119, 120, 121, 122]. MSC‐EVs are very widely researched and reported, likely due to their relatively high availability/accessibility, established guidelines by International Society for Cell & Gene Therapy (ISCT) and the International Society for Stem Cell Research (ISSCR) for cell characterization, and previous clinical experience with MSC cell therapies. Both MSC‐EVs and P‐EVs have extensive preclinical activity demonstrations (immunomodulation, pro‐angiogenic signaling, cytoprotection, wound repair), and are increasingly represented in early‐stage clinical trials. Other sources of EVs have been explored in pre‐clinical studies, including other cultured cells (induced pluripotent stem cells (iPSCs), immune cells, endothelial cells, etc.), as well as plasma/serum, milk, and plant extracts [123, 124, 125]. Each source material comes with different EV yields, co‐isolates, and scalability challenges. This section briefly highlights the therapeutic potential of each source and describes the processing challenges they present.

#### 3.1.1 Cell Culture‐Derived EVs

Among cultured sources, stem‐cell–derived EVs (from embryonic stem cells, induced pluripotent stem cells, and adult MSCs derived from bone marrow, adipose tissue, and umbilical sources) have the broadest evidence base. For example, Adamiak and colleagues demonstrated that EVs derived from iPSCs improved cardiac function in a mouse myocardial infarction model, with efficacy and safety superior to live iPSCs [123]. Among all stem cell sources, MSC‐EVs have the clearest path to becoming regulated products by building on prior cell therapy experience and ISCT characterization frameworks for the parent cells [19, 46, 124, 126]. Therapeutic EVs in pre‐clinical models have been harvested from many other cultured cell types, such as cardiomyocytes, keratinocytes, fibroblasts, macrophages, and endothelial cells, then assessed in disease models, primarily for tissue repair [44, 124, 126].

Compared with other sources discussed in this section, cell culture‐derived EVs pose three major challenges: relatively low EV production rates, high upstream‐driven heterogeneity, and the requirement for handling large sample volumes. Producing clinically relevant doses requires large volumes of cell‐conditioned medium (CCM), and the use of 3D microcarriers, hollow‐fiber formats, or bioreactors to increase EV production [93, 94, 127]. However, as described earlier, these platforms introduce additional variables that influence EV composition and quality [20]. In addition, cell properties can change over time. For example, MSCs across different cell passages produced comparable particle yields but the EV preparations progressively diminished in pro‐angiogenic potency after passage 4, while higher seeding densities and more frequent medium exchanges increased EV output [92]. Even within the same cell type and cell passage, the cargo of EV preparations is variable and not evenly distributed through all vesicles in a population [23, 101, 128]. Thus, the biological variability inherent to cultured cells constrains process standardization and highlights the need for consistent upstream control. The requirement to process large volumes of CCM also creates additional challenges. UC, although prevalent in research, does not scale effectively for bioreactor throughput. Consequently, tangential flow filtration (TFF) has emerged as a preferred alternative, offering closed, continuous, and scalable concentration and diafiltration workflows that can be combined with other isolation methods such as SEC [93, 129]. TFF implementation has typically been more challenging to implement in basic research settings compared with UC, SEC, affinity, or precipitation approaches, which benefit from small‐scale commercially available kits. However, there are recently commercially available benchtop TFF devices, such as Sartoflow (Sartorius) which aim to simplify the process for basic research laboratories. Nevertheless, TFF still relies on separating particles by size and is not suitable for fluids containing abundant particles in the EV size range.

#### 3.1.2 Blood‐Derived EVs

Blood is an attractive source of EVs because it already serves as the basis for many approved clinical products, such as packed red cells, plasma, and platelet transfusions. In fact, EVs are abundant in these blood products, which are already administered to patients, and thus they are well‐positioned for clinical translation [130, 131]. Blood contains a heterogeneous mixture of EVs originating from blood cells (primarily erythrocytes), endothelial cells, and other organs and tissues, although EVs are still greatly outnumbered by NVEPs [132, 133, 134]. It should be considered that whole blood, plasma, serum, and purified blood products (e.g., packed erythrocytes, platelet concentrates) are distinct source materials with unique requirements for EV isolation [135]. For example, purified platelet concentrates are enriched in platelets and often contain a small amount of plasma or platelet additive solution as a plasma substitute [26].

In terms of clinical efficacy, autologous serum EVs have been explored in a small pilot case‐control study in refractory chronic venous ulcers, which reported improved wound metrics compared to sham treatments [136]. Human donor plasma EVs have also been shown to exhibit therapeutic activity in pre‐clinical studies. Plasma EVs reduced myocardial ischemia–reperfusion injury (linked to miR‐486/PTEN signaling and HSP70/TLR4 signaling) in both rodent and canine models and, in rodents, human plasma EVs mitigated intracerebral haemorrhage severity via miR‐25‐3p [125, 137, 138]. While autologous serum or plasma treatments would be advantageous for reducing immunogenicity, EV quality appears to be variable among donors, with notable differences in protective functions between EVs from young/old donors, as well as between healthy/diseased donors [139, 140, 141, 142]. Thus, it is plausible that, for example, the elderly patients with chronic ulcers could have benefited more from allogeneic plasma EVs sourced from young healthy donors. However, the optimal composition of these EV preparations for therapeutic effects has not yet been defined.

Blood product‐derived EVs present several processing‐related challenges. Plasma and serum are complex biological fluids with total protein concentrations ranging from 60–80 mg/mL. Albumin is the most prevalent protein, accounting for approximately 50%–60% of the total, followed by globulins, transferrin, and coagulation factors such as prothrombin and fibrinogen [135, 143]. Plasma and serum contain abundant lipoproteins (HDL, LDL, and VLDL) and protein complexes whose sizes and buoyant densities (in g/cm^3^) overlap EVs; thus, they are present in every blood‐derived EV preparation [135, 144, 145]. It has been estimated that ∼99.9998% of particles in the 10–1000 nm range in blood are lipoproteins and only 0.0002% are EVs [133]. However, it is not clear whether these NVEPs are solely contaminants in EV preparations, since physically linked EV‐lipoprotein complexes have been reported by single particle analysis [146], and these NVEPs can have biological functions; for example, HDL particles carry miRNAs which suppress endothelial cell ICAM‐1 [147]. Thus, these co‐isolates are potentially active participants in the observed effects of blood‐derived EV preparations and can affect apparent isolation yield, measured dose, and functional readouts. As a result, it is difficult to establish the specific activity of the vesicles alone. Accordingly, orthogonal quantification of co‐isolates (e.g., apolipoproteins and albumin) and methods that better distinguish vesicles from similarly sized NVEPs should support both product characterization and functional interpretation. For example, a recent study demonstrated that top‐loaded density gradient separation isolated EVs with a total protein concentration 24 000‐fold lower than whole plasma protein, removing albumin and apolipoprotein and enriching CD63‐positive vesicles [22]. Since co‐isolates may be unavoidable with current isolation techniques, the practical aim should be to achieve consistency of blood‐derived EV preparations, with defined CQAs for acceptable concentrations of EVs, NVEPs, and other proteins.

#### 3.1.3 Milk‐Derived EVs

Milk is one of the most EV‐rich biofluids (∼10^1^ ^2^ vesicles per mL by NTA), making it an attractive source for low‐cost, high‐yield EV preparations [148]. The protein composition of milk‐derived EV (MEV) preparations is influenced by the host species, age, diet, and downstream purification processes. For example, bovine milk contains higher levels of milk fat globule membrane proteins, whereas human MEVs are comparatively enriched in HSP70. In addition to proteins, MEVs carry a wide range of metabolites, including amino acids, B vitamins, and nucleotides, which contribute to their reported anti‐inflammatory and gut‐barrier‐supporting properties [149, 150]. Most research work has explored oral dosing to act locally in the gut, influencing barrier integrity, immune responses, and the microbiome. For example, a landmark study showed that orally administered bovine MEVs restored mucus/epithelial/immune barriers and improved outcomes in diet‐induced nonalcoholic steatohepatitis (NASH) models [151]. Positive effects of MEVs have also been reported in animal models of arthritis, bone growth, repair, and mineral density, as well as in ulcerative colitis and cardiac fibrosis [151, 152, 153, 154, 155]. Oral effects of natural/unmodified MEV administration on cancer have also been investigated, with mixed results. One study intriguingly showed a reduction in primary breast and colorectal tumors, but accelerated metastasis in mice [156]. Since MEVs are food‐derived and orally tolerated, they are also being explored as carriers for small molecules and nucleic acids. For example, Zhang and colleagues demonstrated that MEVs could deliver anti‐TNFα siRNA across intestinal barriers, reducing symptoms in a mouse model of inflammatory bowel disease [157]. Similarly, a 2021 study showed that exogenous miR‐148a‐3p could be loaded into MEVs and subsequently delivered into hepatocytes and intestinal epithelial cells, affecting cell gene expression in these cells [158]. Other groups have also reported the uptake of MEVs and the delivery of labeled EV RNAs to the brain in mice and pigs; however, the quantitative delivery remains unclear [159]. As such, MEVs appear to have some potential as drug delivery vehicles but are not as well researched as cell and blood‐derived EVs. Most studies of MEV remain small‐scale and have relied on sub‐optimal EV tracking methods.

Although abundant in EVs, milk presents several processing challenges. Raw milk is a fat‐rich emulsion containing two major particulates, casein micelles (50–500 nm) and milk fat globules, MFGs (100–1500 nm), which both overlap in size range with EVs [148]. Although MFGs are larger than most EVs, they can be encapsulated by EVs or fragmented into EV‐sized particles during processing [110, 160, 161]. MFGs can be removed by low‐speed centrifugation, and casein micelles can be partially removed using EDTA/citrate chelation, acid (pH ∼4.6) precipitation, or enzymatic approaches. Although these treatments can remove the contaminants, they may also alter EV integrity. For example, acidification is fast and effective for casein clearance but can partially degrade EV‐surface proteins and alter epitope detectability [110, 162, 163]. Comparative studies in human and bovine milk confirm that the choice and severity of casein‐removal conditions materially change EV yield, marker recovery, and co‐isolate profiles, so these steps must be treated as critical process parameters with orthogonal verification (e.g., EV markers alongside β‐casein/MFG signatures) [110, 162]. Thus, the major processing challenge for isolating milk EVs is balancing the removal of non‐EV contaminants while preserving EV integrity. Processing challenges and the presence of non‐EV components may also have influenced the MEV biodistribution and targeting studies mentioned earlier.

#### 3.1.4 Plant‐Derived Nanovesicles

Plant‐derived nanovesicles (PDNVs) are often obtained by homogenization, and thus, we avoid referring to them as EVs. PDNVs have been explored as therapeutics due to the abundance of bioactive compounds found in plants, such as flavonoids, antioxidants, triterpenoids, and polyphenolic compounds, which are generally absent from mammalian EVs [164]. PDNV lipid bilayers are composed mainly of phosphatidic acid (PA), phosphatidylethanolamine (PE), phosphatidylcholine (PC), digalactosyl diacylglycerol (DGDG), and monogalactosyl diacylglycerol (MGDG), unlike mammalian EVs, which are enriched in cholesterol, glycolipids, ceramide, and phosphatidylserine [165]. The composition of PDNV preparations varies across plant species, and many have been explored for therapeutic uses in mammals [166]. Similar to MEVs, PDNVs are often administered orally with the intention of targeting the digestive system. A recent study found that PDNVs from *Centella asiatica* contained a rich cargo of RNAs, lipids, proteins, and phytochemicals, which had potent therapeutic effects in a mouse colitis model [167]. The PDNVs reduced inflammation by modulating the gut microbiome with anti‐inflammatory effects comparable to those of a clinically used medication, 5‐aminosalicylic acid. Other studies have shown that PDNVs prepared from ginger, grapefruit, grape, and broccoli offer protection in rodent models of colitis [168, 169, 170, 171]. Outside of the gut, red onion‐derived nanovesicles promote macrophage M2 polarization and accelerated wound closure in a full‐thickness skin wound model in mice [172]. and lotus‐derived EVs had anti‐inflammatory, pro‐migratory effects on macrophages and skin keratinocytes [173]. Similar to MEVs, PDNVs have been explored as drug delivery vehicles. Wang and colleagues showed that grapefruit‐derived NVs could incorporate methotrexate, increasing therapeutic efficacy in a mouse colitis model and reducing systemic toxicity compared to the free drug [169]. Interestingly, the safety and efficacy of PDNVs appear to vary between delivery routes. For example, intravenous tea‐leaf vesicles triggered hepato‐renal toxicity and immune activation, whereas oral dosing was tolerated and effective [174].

In terms of sample processing, PDNVs can be categorized into secreted EVs and a broader category of nanovesicles. For both preparations, the plant cell wall presents a major processing challenge. To isolate true secreted EVs, apoplastic fluid must be extracted, while avoiding tissue/cell disruption, which produces large amounts of cytoplasmic/membrane vesicles and other fragments [175]. This is typically accomplished by vacuum infiltration followed by gentle centrifugation, but EV yields are modest. Most high‐yield workflows instead homogenize/juice tissues and enrich the resulting vesicles. These PDNV preparations carry matrix co‐isolates (cell‐wall polysaccharides such as pectin/cellulose, starch, chlorophyll, and other metabolites) and vesicle mimetics from disrupted membranes. Because the composition of the PDNV preparation depends on mechanical energy, pH, temperature, and clarification sequence, there is greater batch‐to‐batch and source‐to‐source variability (species, cultivar, tissue, growth stage), and the field still lacks standardized operating protocols [165, 176].

Cargo stability is a second processing challenge of PDNVs. Plant extracts contain abundant RNases that rapidly degrade extravesicular RNA unless it is protein‐shielded, implying that insufficient control of temperature, time, and inhibitors can reshape the nucleic‐acid fraction of PDNV cargo during processing. [177]. Likewise, many reported PDNV bioactivities are attributed to phenolics and other phytochemicals that are pH‐ and oxidation‐sensitive, reinforcing the need for suitable buffers and cold handling in vesicle workflows [178, 179]. As such, PDNVs possess interesting properties for exogenous drug delivery or as carriers of natural substances, but they remain further from clinical translation than other EV sources. Illustrating this, one clinical trial (NCT03493984) for the use of ginger and aloe‐derived vesicles in polycystic ovary syndrome (PCOS) was registered but later withdrawn with no patients recruited.

### 3.2 EV Manufacturing in Clinical Translation

As discussed in earlier and later sections, the challenges associated with isolating and characterizing EV preparations make downstream applications difficult. Thus, continual innovation of new technologies that boost the translational capabilities of such processes is critical. Table 1 summarizes selective EV‐based therapeutics in clinical or advanced preclinical development discussed in this review. One illustrative example is Lonza's entry into the EV field through the acquisition of Codiak BioSciences’ cGMP exosome manufacturing facility in November 2021 [180]. Lonza has positioned its Xcite EV platform as an engineered‐vesicle technology supported by prior clinical and preclinical experience generated using Codiak's assets (exoSTING, exoASO‐STAT6, and Exo‐IL‐12) [181, 182]. The Xcite EV technology uses two EV‐enriched proteins, PTGFRN and BASP1, as scaffold proteins to improve EV loading with exogenous molecules. PTGFRN anchors payloads to the EV surface, while BASP‐1 preferentially directs them into the vesicle lumen. By fusing therapeutic molecules to these scaffolds via engineered plasmids and expressing them in producer cells (e.g., HEK293 or MSCs), the system enables higher‐density loading of surface‐displayed or intraluminal cargo into secreted EVs [183].

**TABLE 1:** Selected EV‐based therapeutics in clinical or advanced preclinical development discussed in this review. This table highlights selected examples discussed in this review and is not intended to be an exhaustive list of EV clinical programs. For a comprehensive overview of EV clinical trials, see Mizenko et al., 2024 (J Extracell Vesicles).

Product	Company	EV source	EV type	Indication(s)	Stage	Trial ID(s)	Key observations
**ExoFlo**	Direct Biologics	BM‐MSC conditioned medium	Natural (unmodified)	COVID‐19 ARDS; Crohn's disease; Ulcerative colitis	**Active** Phase 3	NCT05354141 (EXTINGUISH‐ARDS); NCT04493242 (EXIT‐COVID19)	First widely publicized EV therapeutic in RCTs. Safety confirmed. Mortality benefit in subgroup. Criticized for limited transparency in dose, purity, and CQA reporting.
**PEP^TM^ (Purified Exosome Product) / PEP Biologic^TM^**	RION / INTENT Biologics (Mayo Clinic spin‐out)	Human platelet‐derived EVs	Natural (unmodified)	Diabetic foot ulcers; knee osteoarthritis; myocardial infarction (MI); dermal aesthetics	**Active** Phase 2 (DFU); Phase 1b (knee OA); Phase 1 (dermal)	NCT04327635 (MI); NCT06429033 (dermal); NCT06793748 (ulcer); NCT06319287 (DFU); NCT06463132 (OA)	Shelf‐stable lyophilized powder (24‐month RT shelf life, up to 2 × 10^1^ ^2^ particles/vial). FDA Fast Track designation for DFU (Jan 2026) via INTENT Biologics. Phase 2 DFU trial (n=59) showed 54% healing response vs. 25% standard care (per company report). Phase 3 planned for 2026. Lonza cGMP manufacturing partnership (Sept 2025).
**iExoKrasG12D**	PranaX Corp. (licensed from MD Anderson)	Engineered BM‐MSC EVs	Engineered (KRAS G12D‐targeted siRNA)	Pancreatic ductal adenocarcinoma	**Active** Phase 1	NCT03608631 (iEXPLORE)	Well tolerated; no dose‐limiting toxicities. Evidence of target engagement and immune modulation including increased CD8^+^ T‐cell infiltration.
**AB126**	Aruna Bio	Neural stem cell‐derived EVs (proprietary cell line)	Natural (unmodified)	Acute ischemic stroke; ALS (preclinical); Alzheimer's Disease; Parkinson's Disease, Huntington's Disease; prion disease	**Active** Phase 1b/2a (IND cleared Jan 2024)	Pending (trial initiation planned)	First EV IND cleared by FDA for a neurological indication. Claimed innate BBB traversal and anti‐inflammatory, neuroprotective properties. In‐house GMP manufacturing. Fundraising ($15 M) underway to initiate first‐in‐human dosing.
**EXOB‐001**	EXO Biologics	MSC‐derived EV	Natural (unmodified)	Bronchopulmonary dysplasia in premature infants	**Active** Phase 1+2	2022‐500293‐34‐01 (EVENEW)	The first MSC‐EV therapeutic trial to be approved by the EMA. Intratracheal administration. Currently
**CPC‐EVs (SECRET‐HF)**	Academic consortium	iPSC‐derived cardiac progenitor cells	Natural (unmodified)	Non‐ischemic dilated cardiomyopathy	**Active** Phase 1	NCT05774509	Assessing safety of iPSC‐derived cardiac progenitor EVs. Represents academic‐led translation pathway.
**Plexaris**	ExoPharm (now Entropy Neurodynamics as of Nov 2025)	Allogeneic platelet concentrates	Natural (unmodified)	Wound healing	**Discontinued** First‐in‐human (completed)	ACTRN126200 00944932 (Plexoval II)	Isolated by LEAP. 100 µg in 340 µl well‐tolerated for intradermal injection. Plexaris product wound down. LEAP technology available for licensing.
**exoSTING^TM^**	Lonza (Xcite platform; ex‐Codiak)	Engineered HEK293	Engineered (PTGFRN scaffold, luminal CDN loading)	Advanced solid tumors	**Inactive** Phase 1	NCT04592484	>100‐fold greater intratumoral potency vs. free cyclic dinucleotide STING agonist in preclinical models. Increased tumor retention, reduced systemic cytokine exposure.
**Exo‐IL‐12^TM^**	Lonza (Xcite platform; ex‐Codiak)	Engineered HEK293	Engineered (PTGFRN scaffold, surface IL‐12 display)	Solid tumors	**Inactive** Preclinical (NHP)	—	10‐fold higher tumor retention of IL‐12 vs. recombinant protein in NHP. No detectable systemic exposure after intratumoral injection. Stimulates IFN‐γ secretion from tumor‐resident immune cells.

Systemic delivery of free cyclic dinucleotides (CDNs), which act as STING agonists, has shown dose‐limiting inflammatory toxicities and limited clinical efficacy, motivating strategies that localize their effects to the tumor microenvironment [184]. Codiak's exoSTING, an EV formulation of cyclic dinucleotide STING agonists, demonstrated >100‐fold greater intratumoral potency relative to free CDN in preclinical models, with increased tumor retention and reduced systemic cytokine exposure, and was evaluated in a Phase I clinical study (NCT04592484) [185]. For the Exo‐IL‐12 product, functional IL‐12 is displayed on the EV surface, which stimulates tumor‐resident immune cells to secrete IFN‐γ, driving an anti‐tumor immune response. Non‐human primate studies have reported 10‐fold higher tumor retention of IL‐12 with Exo‐IL‐12 compared to the recombinant IL‐12 protein, with no detectable systemic exposure after intratumoral injection [186]. Beyond these early engineered EV programs, Lonza has continued to expand its EV biomanufacturing capabilities, announcing in late 2025 a cGMP manufacturing partnership with Rion for their platelet‐derived EV therapeutics, demonstrating sustained industrial interest in EV drug development [65]. In parallel, academic‐industry translation is also progressing. In March 2025, MD Anderson Cancer Center granted PranaX Corporation a license to a patent portfolio covering exosome manufacturing, engineering, and therapeutic use [187]. In a Phase I study (iEXPLORE, NCT03608631), engineered BM‐MSC EVs bearing KRAS G12D‐targeted siRNA (iExoKrasG12D) were shown to be well tolerated, with no dose‐limiting toxicities, and showed evidence of target engagement and immune modulation (including increased CD8^+^ T‐cell infiltration) in patients with advanced pancreatic ductal adenocarcinoma [188, 189]. In tandem, developed first at the University of Georgia's Regenerative Bioscience Center, AB126 – a neural stem cell‐derived EV—was the first exosome IND to be cleared by the FDA for acute ischemic stroke, and pre‐clinical studies for myotrophic lateral sclerosis, and Alzheimer's disease. At the time of writing, Aruna Bio, AB126's company, is fundraising $15 M to initiate clinical trials with first‐in‐human dosing [180, 190, 191], in addition to establishing patents for EV‐based delivery to neural targets. On the global scale, trials like that of EVENEW, which test the safety of MSC‐derived EV in infants experiencing bronchopulmonary dysplasia and is the first of its kind to be approved by the EMA [192, 193], demonstrate an interest in better understanding EV's efficacy in vulnerable populations. Together, these examples illustrate how EV biology, molecular engineering, and industrial‐scale manufacturing are converging toward more standardized, drug‐like EV therapeutic products.

### 3.3 Emerging Isolation Methods

Previously, we have discussed well‐established EV isolation methods, including UC, immunoaffinity capture, ultrafiltration, precipitation, and SEC [103]. TFF and anion‐exchange chromatography have also been well described by other articles [194, 195, 196]. In general, mainstream approaches such as ultrafiltration are widely accessible and can accommodate larger sample volumes, but they typically provide coarser enrichment and remain susceptible to co‐isolated particles. In contrast, emerging fractionation approaches can offer higher‐resolution separations and improved control over size‐defined subpopulations, but often operate at lower throughput (e.g., only a few mL per run) and require specialized platforms. Therefore, this update highlights three novel isolation methods: asymmetric flow field‐flow fractionation (AF4), EV‐Osmoprocessor (EVOs), and deterministic lateral displacement (DLD). These methods address major challenges in EV isolation, such as achieving higher throughput and higher purity, and separating particles with greater precision.

#### 3.3.1 Asymmetric Flow Field‐Flow Fractionation (AF4)

AF4 separates nanoparticles in a thin, flat channel using two perpendicular flows: a forward channel flow and a cross flow through a semi‐permeable membrane [197]. The cross flow pushes particles toward the membrane, while Brownian motion drives them back into the channel; the equilibrium between these forces positions particles at different heights depending on their diffusion coefficients. Since laminar flow velocity increases with height, smaller, more diffusive particles elute earlier, while larger, less diffusive particles elute later. Advantages of AF4 include nanometer‐scale resolution, label‐free, rapid, and gentle isolation. Due to its high resolution, AF4 can be used to resolve distinct small EV subsets by size, which may address questions around EV heterogeneity and sub‐populations outlined in Section 2.1. However, the method requires specialized instrumentation and technical expertise to optimize parameters such as cross‐flow intensity, channel geometry, and membrane type. Samples must also be pre‐cleared and concentrated before injection. Finally, because AF4 is size‐based, it cannot inherently separate lipoproteins from blood‐derived samples, which overlap in hydrodynamic size with EVs. To address this issue, a recent study combined AF4 with a density cushion UC pre‐step, which effectively removed the bulk of soluble proteins and lipoproteins from plasma and serum samples prior to fractionation [144]. When the enriched pellets were run through AF4, EVs were cleanly resolved into a late‐eluting peak, with minimal co‐isolation of ApoA1‐ or ApoB‐positive particles, compared to SEC (qEV system). This combined strategy enabled the recovery of high‐purity blood EVs, suitable for downstream proteomic and RNA profiling. Based on this high isolation purity, the study identified protein markers (MYCT1, TSPAN14, etc.) that were more specific to plasma EVs than typical markers (CD9/CD81 etc.). Another strategy, beyond density‐cushion pre‐enrichment, is to add an orthogonal electric‐field separation step. For example, offline AF4–capillary electrophoresis separates EVs first by hydrodynamic size and then by electrophoretic mobility/surface charge, enabling serum EVs to be distinguished from similarly sized contaminants, including LDL particles that co‐elute in AF4 alone [198, 199]. Together, AF4 is a highly promising emerging technique, but wider adoption will require simplification into standardized, user‐friendly platforms rather than custom instrument setups.

#### 3.3.2 EV‐Osmoprocessor (EVOs)

EVOs use a high‐osmolarity polymer solution to create controlled osmotic gradients across a permeable membrane [200]. Based on the osmotic pressure difference, water and small proteins (such as albumin) pass through the membrane and are removed from the sample, while larger particles, such as EVs, are retained. By removing water, the process also reduces sample volume and concentrates substances that exceed the membrane molecular weight cut‐off threshold. For example, EVOs passively achieved a 50‐fold volumetric reduction (10 mL sample to 200 µL processed specimen) of MSC‐conditioned medium, while removing 99.7% of albumin within two hours. The EVOs process alone achieved an EV preparation particle‐to‐protein ratio of ∼1 × 10^7^ particles/µg protein, representing a significant purity enhancement compared to unprocessed cell culture media. EVOs were also used as a simple upstream step to concentrate CCM samples prior to SEC, increasing SEC processing capacity 60‐fold and further improving the particle‐to‐protein ratio to ∼1 × 10^9^ particles/µg protein. The EVO process is scalable, fast (∼2 h), requires minimal user intervention, and is particularly suitable for concentrating large sample volumes before additional purification.

#### 3.3.3 Deterministic Lateral Displacement (DLD)

DLD is a microfluidic size‐sorting method that uses an array of microscopic obstacles to continuously separate particles by size as fluid flows through the array [201]. In a DLD device, rows of pillar obstacles are slightly offset from the previous row, creating streamline bifurcation. Particles below a certain critical size follow the flow streamlines in a zigzag path, but larger particles are physically bumped into an adjacent streamline at each obstacle, causing a lateral displacement across the array. The spacing determines the critical diameter and offset geometry of the obstacles. The channel design parameter and the flow rate are key factors for tuning the cutoff size and throughput of DLD. A recent nanoscale DLD device can sort small EVs from larger vesicles or debris with high precision. For example, Wang et al. developed a thermally oxidized nonuniform DLD array that created an effective cutoff at 200 nm for EV separation [202]. In their design, asymmetrically tapered nanopillars allowed smaller EVs to pass through undeflected while diverting larger contaminants. Using cell culture media spiked with 600 nm beads, the study demonstrated a significant improvement in EV purity after passing through the DLD chips, with EV morphology maintained. The advantages of DLD include label‐free operation, passive separation, tunable cutoffs, and relatively high EV yields with intact cargo and high consistency [203]. However, DLD cannot remove similarly sized NVEPs from a preparation, and total throughput remains limited. For example, Smith and colleagues demonstrated processing at 15 µl/min, although this was offset by higher yields and EV concentration compared to SEC.

### 3.4 EV Storage

Whether EVs are isolated and characterized for research or therapeutic purposes, their storage conditions must be addressed. This includes long‐term shelf‐life temperature, various stability‐indicating analytical assays, and acceptance criteria used to determine stability, as well as post‐storage handling operations. For therapeutics, this also includes considering their in‐use handling and stability conditions. The frozen, −80°C condition is the most practical baseline for long‐term storage; however, freeze‐thaw cycles have been shown to alter EV preparations by promoting fusion/aggregation, resulting in decreased particle counts, increased particle size, reduced RNA content, and lower potency [204]. Therefore, single‐use aliquots are preferred, such as in analytical testing studies, wherein the best practice is to minimize sample handling and use multiple vials/containers, or subaliquots, instead of refreezing and thawing EV bulk materials. The storage medium (formulation) is also critically important for EVs being handled in the liquid state, or for 2–8°C refrigerated storage conditions after thawing from the frozen state, or short‐term storage at −20°C. For example, a well‐cited study demonstrated that storage in PBS alone is rapidly (beginning within minutes) detrimental to the recovery rate of MSC‐EV and HEK293T‐EV preparations due to aggregation and plasticware adsorption [205]. This resulted in ∼50% lower NTA particle counts, lower RNA content, and reduced cell uptake after 6 months storage at −80°C, and 70%–90% loss when stored at −20°C. Supplementing PBS with human albumin (0.2%) and trehalose (25 mM) and storing at −80°C preserved recovery and protected EV RNA content from degradation [205]. Sucrose buffers and surfactants such as polysorbate 80 have also been shown to reduce EV aggregation after freeze‐thaw [204]. Lastly, dry storage after lyophilization can be used to extend the long‐term shelf life of EVs. Trenkenschuh and colleagues compared freeze‐dried and wet storage (control condition) of human B‐lymphoblastoid cell EVs and bacterial outer membrane vesicles (OMVs) [206]. They found that freeze‐dried EVs, formulated in 10 mM Na‐ or K‐phosphate buffer (pH 7.4) containing 5% sucrose and 0.02% poloxamer 188, retained their proper particle size and concentration for at least 6 months at 40°C. Interestingly, however, EV enzymatic cargo activity began to decline after one month. This again highlights the importance of employing a functional analysis amongst the characterization assays, rather than relying solely on particle count and size measurements to determine a laboratory shelf life. MISEV23 does not provide specific storage guidelines but recommends characterizing EV preparations before and after storage [3]. It should be noted that standard bulk EV characterization methods (particle counts, protein concentration, marker proteins, etc.) are limited in their ability to detect changes in the EV preparation. Therefore, potency assays using predetermined acceptance criteria should be used to verify biologic activity for research materials, at a minimum [207].

It is also important to emphasize that the overall analytical testing scheme that supports stability assessments for any type of therapeutic material administered to humans, including EVs, is significantly more comprehensive and detailed than what can be learned from particle count, concentration, and potency assessments alone. This more comprehensive analytical scheme and its considerations are discussed in more detail in Section 4.2.

## 4 Characterization of EV Preparations

After isolation, an EV preparation must be characterized, including the vesicles and co‐isolates. Again, for therapeutic translation of EV to humans, it is important to establish precisely what is being administered to patients, and this may be required by regulatory agencies governing the administration of therapeutics to patients. A more in‐depth discussion on those analytical requirements is presented in Section 4.2. However, no matter the application of EVs, routine bulk/population‐sample analytical tools, including but not limited to NTA for size/count, immunoblots/PCR for composition, and transmission electron microscopy for morphology, are informative but still inherently limited in their ability to provide information about the overall quality of the materials. These techniques average over heterogeneous mixtures or characterize only a small fraction of the entire EV preparation and are subject to sensitivity and resolution limits. The minimum detectable size and refractive‐index assumptions vary by platform, which alone can shift reported concentrations by several orders of magnitude [208]. Consequently, accurate and reproducible characterization of EVs requires multiple complementary measurements, beginning with fundamental assessments of particle size and concentration.

Current practice for EV size measurements relies heavily on light scattering detection methods that infer particle size distributions from Brownian motion or scattering intensity. Yet, as emphasized by MISEV23, these measurements should be interpreted within the context of their methodological constraints and complemented with orthogonal approaches [3]. Among the most widely adopted techniques are DLS and NTA, which illustrate the practical considerations and trade‐offs in EV measurement. DLS measures fluctuations in the intensity of scattered light to derive a correlation function that reflects the ensemble Brownian motion of all particles in suspension, from which it estimates the hydrodynamic radius and reports parameters such as the Z‐average diameter and polydispersity index (PDI) [209]. Because scattering intensity scales with the sixth power of particle diameter, a small proportion of larger particles or aggregates can dominate the signal, biasing size estimates upward. DLS is rapid, requires minimal operator input, is highly reproducible for near‐monodisperse samples, and has been commonly used to characterize micelles and nanoparticle preparations [210, 211]. However, it does not provide particle concentration and is less well suited for heterogeneous or polydisperse EV preparations. NTA, in contrast, tracks the Brownian motion of individual particles under a laser‐illuminated microscope and calculates hydrodynamic diameters from single‐particle trajectories. The approach provides both a number‐weighted size distribution and a direct measure of particle concentration (particles/mL), making it more suitable for complex biological fluids where vesicles coexist with non‐vesicular nanoparticles and size heterogeneity is high [212, 213]. However, NTA alone cannot distinguish between EVs and other particles, and its sensitivity typically extends down to ∼50 nm, with resolution decreasing for particles below ∼80 nm and at very high concentrations where particle overlap confounds tracking. More modern NTA systems, such as the Malvern Nanosight Pro, can combine NTA with fluorescence detection to distinguish labeled subpopulations based on EV surface proteins or cargo. However, compared to DLS, NTA requires more hands‐on optimization, such as focus adjustment, sample dilution, and video capture settings, and is more time‐consuming, with greater operator‐to‐operator variability [214]. For EV research, it is therefore recommended to use both methods in a complementary manner. Nevertheless, even with these techniques, reported EV metrics remain strikingly inconsistent across studies, underscoring the importance of methodological transparency and orthogonal correlation of methods to provide a holistic understanding of material quality.

**SCHEME 2:**
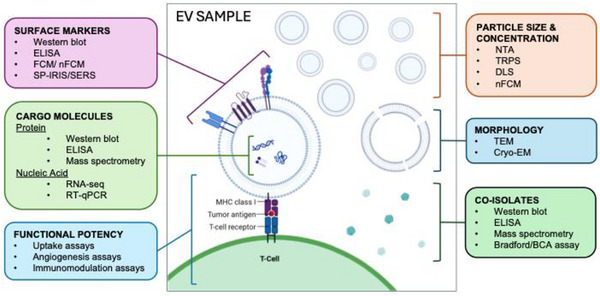
Orthogonal Analytical Framework for Characterizing EV Preparations.

Scheme 2 summarizes a set of orthogonal analytical approaches that together define the quality of an EV preparation (as a drug‐like mixture) while also enabling measurements at the single‐EV level and linking composition to functional potency. At the preparation level, particle size and concentration can be benchmarked using complementary sizing/counting platforms (NTA, TRPS, DLS, and nano‐flow cytometry (nFCM)), while morphology and vesicular integrity can be verified by TEM or cryo‐EM to confirm bilayer structures and assess heterogeneity. In parallel, the burden of co‐isolated proteins can be quantified using BCA and characterized by western blot, ELISA, and mass spectrometry, supporting impurity profiling and lot‐to‐lot comparability. To establish EV identity and composition, EV surface markers and cargo molecules can be assessed using orthogonal assays—western blot/ELISA for targeted proteins, nFCM and SP‐IR for phenotyping and classification of vesicle populations, SERS for spectral fingerprinting, and RT‐PCR/RNA‐seq for nucleic acid cargo profiling. Finally, these physicochemical and molecular readouts should be connected to potency‐relevant functions using standardized in vitro assays such as EV uptake/internalization, angiogenesis, and immunomodulation assays, enabling a multi‐layered characterization framework in which particle metrics, identity/purity markers, and cargo signatures are jointly interpreted against functional performance.

### 4.1 New Characterization Techniques

Research is now moving towards high‐throughput single‐particle methods that can better describe EV heterogeneity. These include flow‐based approaches (nano‐flow and imaging flow cytometry), multiplexed surface capture (e.g., interferometry/ExoView), and targeted single‐EV assays that co‐localize surface and cargo molecules. These tools can reveal which markers co‐localize on the same vesicle, where those signals are located (e.g. EV corona, membrane, or vesicle lumen), and how EV subpopulations shift in response to biological or processing changes [215, 216]. In the following sections, key advances will be highlighted.

#### 4.1.1 Nano‐Flow Cytometry (nFCM)

nFCM adapts flow cytometry for analyzing particles <200 nm in si, such as EVs, viruses, and nanoparticles. This is accomplished by combining low‐noise optics (often photon‐counting detectors), low flow rates, and calibration to translate side‐scatter and fluorescence into particle properties [128, 217]. In practice, modern nFCM platforms can detect individual EVs down to ∼40 nm (instrument‐ and sample‐dependent) and read out size/side scatter plus one or more fluorescence channels per event, enabling quantitative counting and phenotyping rather than bulk averages. These capabilities have been demonstrated across both lab‐built and commercial systems and verified in head‐to‐head studies against nano‐imaging approaches. For example, nFCM enabled the detection of rare disease‐related EVs and successfully distinguished between genuine EVs from protein aggregates in UC preparations [218, 219]. nFCM also enables label‐based detection of EV subpopulations using fluorescent antibodies against target protein markers. In addition, adapted high‐resolution flow sorters (nanoFACS) can enable fluorescence‐based sorting of EV subpopulations for downstream characterization [220]. This directly addresses a key limitation of non‐specific particle detection methods such as NTA, which have difficulty distinguishing vesicles from protein aggregates [221]. This also enables the resolution of EV subpopulations and marker co‐expression patterns. Work by Andronico and colleagues showed that correcting for vesicle transit time in multicolor flow improved true on‐particle co‐localization (e.g., CD63/CD81) versus standard cross‐correlation, enabling rigorous per‐vesicle phenotyping [222]. Additionally, Mizenko and colleagues showed that tetraspanin markers (CD9/CD63/CD81) were not uniformly expressed within EV preparations from human serum, cultured MSCs, and an ovarian cancer cell line, again illustrating heterogeneity and the need for orthogonal measurements [223].

As nFCM is becoming more widely used in basic EV research, ISEV has published guidelines (MIFlowCyt‐EV) for conducting and reporting EV flow cytometry experiments, as experimental parameters greatly influence reported results [224]. A multi‐center comparison of commercial high‐sensitivity flow cytometers versus a custom single‐molecule system showed that reported EV concentrations and surface‐marker copy numbers depend strongly on each instrument's limit of detection (LOD). Commercial instruments (CytoFLEX and CellStream) detected only the brighter and larger EV subsets, which accounted for ∼1%–6% of the events detected by the single‐molecule cytometer. As such, the commercial instruments also produced higher apparent particle sizes and label copy numbers. However, when results were filtered to a common LOD (≈10 phycoerythrin molecules/80 nm), inter‐platform agreement improved markedly, highlighting the necessity of defining and reporting LODs when comparing nFCM datasets [225]. This aligns with MIFlowCyt‐EV recommendations to specify the analyzed population, rather than implying unbiased sampling [224]. Calibration of nFCM with size and fluorescence standards is essential, and sample dilutions must be carefully prepared to avoid “swarm” effects, in which multiple particles appear as a single event. Label specificity is also a key variable, as some commonly used fluorescent dyes lack specificity (such as MemGlow, which shows affinity for staining very low‐density lipoprotein (VLDL) particles) or can self‐generate aggregates or form micelles that produce misleading results [226, 227]. Recently, results from a multinational academic and research collaboration showed that orders‐of‐magnitude variability in results between flow cytometers could be greatly reduced by proper calibration of flow rate [134], fluorescence, and light scattering. Using calibrated flow cytometers, the authors then established reference ranges for blood cell‐derived EVs in human plasma, which is an encouraging step towards achieving standardization.

#### 4.1.2 Interferometry (SP‐IRIS, iSCAT)

Interferometric platforms image individual nanoparticles by detecting changes in reflected or scattered light at a surface, enabling single‐EV counting, sizing, and phenotyping [228, 229]. In single‐particle interferometric reflectance imaging sensing (SP‐IRIS), commercially exemplified by NanoView Biosciences’ ‘ExoView’ products, EVs are captured on antibody microarrays (often anti‐CD9/‐CD63/‐CD81), then quantified label‐free and phenotyped by on‐chip immunofluorescence. SP‐IRIS can analyze minimally processed biofluids, report per‐particle marker co‐localization, and typically resolve vesicles in the ∼50–200 nm range, bringing subpopulation structure into view that bulk tools can miss. Similar to nFCM, SP‐IRIS studies have shown that canonical EV markers are not uniformly expressed: tetraspanins display source‐dependent, non‐overlapping distributions across single EVs, illustrating true biological heterogeneity [13]. This creates an inherent limitation for any tetraspanin capture‐based system, as only the particles that are captured will be measured.

To address this limitation, interferometric scattering microscopy (iSCAT) measures EVs based on interferometric contrast without antibody capture [230, 231]. For example, EVs can be physically immobilized on poly‐L‐lysine–coated substrates, where the positively charged surface facilitates charge–charge interactions with the negatively charged EV membranes, enabling size determination and subsequent fluorescence co‐registration on the same particles [232]. A recent high‐throughput example was able to size EVs from ∼37 to ∼220 nm, distinguish <100 nm subpopulations, and analyze >10 000 EVs in ∼7 min across multiple fields of view, illustrating how label‐free size‐photometry and fluorescence imaging can be combined without capture antibodies. Interferometry can also be combined with mass‐photometry (“interferometric NTA”) approaches that extract size and refractive index distributions from weakly scattering nanoparticles in suspension [233]. This can be used to separate EVs from lipoproteins/protein aggregates when their refractive indices differ. Demonstrations include resolving polydisperse mixtures and biological samples with sensitivity in the ∼10 nm range.

Despite their sensitivity, interferometric approaches generally require careful calibration and clean, low‐background surfaces to distinguish weakly reflected or scattered signals from background noise. Background correction and dark calibration are critical for reliable signal detection in SP‐IRIS and similar platforms [234]. Moreover, although SP‐IRIS can routinely detect EVs ∼50 nm in size, accuracy near that limit is compromised by sample drift, nonspecific binding, and background fluorescence, which must be minimized to avoid over‐ or under‐estimation of vesicle counts. Throughput is also lower than that of nFCM‐based approaches [13].

#### 4.1.3 Raman Spectroscopy

Raman spectroscopy is a label‐free analytical technique that measures the molecular composition of a sample based on the inelastic scattering of light [235, 236, 237, 238]. During measurement, a monochromatic laser is focused onto the sample. Most of the photons scatter elastically through a phenomenon known as Rayleigh scattering, and a small fraction of photons scatter inelastically through a phenomenon known as Raman scattering. The resulting Raman spectrum can be evaluated to extract chemical signatures that reveal the chemical composition of the EV, such as the relative abundance and types of biomolecules present (e.g., lipids, proteins, nucleic acids, carbohydrates) as well as structural features such as bond order, molecular conformations, or presence of specific functional groups [239]. Raman spectroscopy can be used for single‐EV analysis when combined with enhanced platforms, such as surface‐enhanced Raman spectroscopy (SERS) or optical trapping [240, 241]. Single‐particle SERS maps biochemical fingerprints (protein/lipid/nucleic‐acid bands) for thousands of vesicles, revealing subpopulation structures. The approach demonstrated label‐free differentiation, including separating EVs from activated vs. non‐activated blood platelets and EVs/lipoproteins from prostate cancer patients versus non‐cancer controls [242, 243]. Several groups have now demonstrated the ability to distinguish between EVs and lipoproteins based on Raman spectra. Raman spectroscopy can also be used to differentiate between EV populations within a preparation. Penders et al. used single‐particle automated Raman trapping analysis (SPARTA) to distinguish breast cancer‐derived EVs from healthy EVs based on subtle differences in their overall biochemical fingerprints [240]. Thus, Raman is a powerful tool that can measure what components are included in an EV preparation, and attribute those signals to either vesicular or non‐vesicular components. This directly addresses the challenges of EV heterogeneity and standardization between batches mentioned earlier. Limitations of the technique include the relatively low throughput and the high degree of complexity and expense of the equipment. In samples with diverse molecular compositions, such as EV preparations, the resulting Raman spectrum can be complex and varied. Here, multivariate or machine learning analyses can be used for reliable interpretation, although the predictive models may be affected by changes to sample processing and handling [244].

### 4.2 Benchmarking and Critical Quality Attributes

#### 4.2.1 Quality Indicators

EVs as therapeutics, like any therapeutic, are required to follow ICH Guidelines governing quality as it relates to safety and efficacy. Critical Quality Attributes (CQAs) define the expectations surrounding the quality of a drug product. ICH Q8(R2) provides the overall guidelines for CQAs, which are deemed as the physical, chemical, biological, or microbiological properties of a drug product that must be controlled within defined limits to ensure safety, efficacy, and consistent quality [245]. In particular, CQAs are the measurable characteristics of the therapeutic product; importantly, they act as the key link between the process development activities for a therapeutic's production, and its safety and efficacy considerations. Since EV preparations are heterogeneous systems with complex compositions, defining the analytical features that determine identity, purity, potency, and safety cannot be simply extrapolated from other well‐characterized biological therapeutics, such as monoclonal antibodies. Consequently, the specific physicochemical and biological characteristics that must be analytically assessed and controlled are still an evolving topic under active regulatory discussion. This may require the use of multiple assays, orthogonal characterization of identity and purity (including co‐isolates/impurities), and potency assays to support lot release, stability, and comparability.

At present, a comprehensive characterization of EV quality and considerations for CQAs are derived from a set of systematic measurement standards. Efforts informed by the International Council for Harmonisation (ICH) quality framework (e.g., ICH Q6B for specifications of biotechnology‐derived products and ICH Q5E for comparability after manufacturing process changes) and World Health Organization (WHO) biologics standards are beginning to shape how EV CQAs may be assessed, though no EV‐specific harmonization program has yet been formally established. ISEV has also proposed minimal experimental criteria for identity and purity testing to enhance reproducibility across laboratories, providing a foundation for the CQA assessments in a GMP setting. [3] Although MISEV23 provides a framework for disciplined product definition and reporting, it is not a prescription for the analytical development sciences needed for GMP‐based manufacturing processes. MISEV emphasizes transparent reporting of methods, orthogonal identity/purity measurements, assessment of co‐isolates, and the use of relevant functional assays. However, it does not specify product‐specific release criteria or potency assay methods, as these must be determined on a case‐by‐case basis for each indication. Nevertheless, adoption of MISEV recommendations during EV manufacturing will improve the comparability of methods across EV studies and link GLP‐ and non‐GLP‐based analyses in EV clinical and translational science, respectively, thereby providing a stronger basis for CQA justification and GMP regulatory oversight.

Beyond the analytical control considerations discussed in the MISEV23 framework, there is a broader control strategy around CQAs that forms part of a holistic approach for EV manufacture under GMP conditions, requiring a robust quality management system (QMS). While CQAs define what must be controlled to ensure safety and efficacy, a QMS defines how these attributes are achieved consistently across batches, sites, and time. A QMS is a formalized system that documents processes, procedures, and responsibilities to meet regulatory requirements while supporting continuous performance improvement, with ISO 9001 widely regarded as a gold standard framework for quality management [246]. In the pharmaceutical setting, a QMS usually refers to the Pharmaceutical Quality System (PQS) described in ICH Q10 [247], the harmonized quality management system model for the pharmaceutical industry. The PQS is the structured set of policies, procedures, processes, records, and responsibilities that ensure product quality and regulatory compliance across the lifecycle of a therapeutic from development through commercial supply. As an umbrella system, it typically covers document control, training, change control, deviations, corrective and preventive action (CAPA), risk management, audits, and management review, underpinning the entire GMP compliance framework. In practice, a QMS translates CQAs into controlled operations using tools such as standard operating procedures (SOPs) for standardization and technology transfer, design of experiments (DoE) to optimize multi‐variable biological processes, and failure mode and effect analysis (FMEA) to identify process risks and define appropriate controls [246]. Risk‐based approaches consistent with ICH Q9 are particularly relevant for managing batch‐to‐batch consistency in complex biologics [248]. For clinical implementation, EV assays must also satisfy medical laboratory QMS requirements (e.g., ISO 15189), including structured validation of performance characteristics for in‐house methods, verification of commercial kits in the intended setting, and ongoing monitoring via audits and batch acceptance [249]. Integrated quality control across raw material preparation, isolation, characterization, and storage is also critical for maintaining functional integrity, including for plant‐derived EV products [250].

A QMS for cell‐secreted EVs must integrate EV‐specific risks into classical GMP frameworks for biologics. At a minimum, such a system should include a plan to cover the evaluation and control of the following [251]; (1) source cells and materials, including qualified donor/materials, master and working cell banks, traceability, and product characterization including identity, sterility etc.); (2) process definition and validation, encompassing closed or functionally closed culture systems, defined (ideally xeno‐free) media, standardized harvest and validated isolation methods (i.e. ultrafiltration, SEC, or TFF) with demonstrated robustness and virus safety where applicable; (3) in‐process controls, including control of critical process parameters (CPPs) for cell expansion, medium conditioning time, harvest conditions, filtration/concentration, and storage of intermediates with predefined action limits and deviation management; (4) CQA assessments of identity, purity (including co‐isolates/impurities), and potency; (5) GMP facility and documentation controls including GMP‐compliant cleanrooms, appropriate biosafety level classification, environmental monitoring, and equipment qualification tailored to cell and EV handling); (6) product lifecycle management, spanning formulation, storage, and shipment, with attention to sensitivity of EVs to handling, freeze–thaw cycles, and lyophilization; and (7) ongoing risk management, including formal risk assessments for heterogeneity in vesicle populations, batch‐to‐batch variability in cargo, and incomplete understanding of mechanism(s) of action, mitigated through tighter process controls and characterization packages.

#### 4.2.2 Characterization Consideration

Some of the key considerations for EV characterization approaches stem from the typical CQA profiles of other biologics, notably their intrinsic purity profiles, which include assessments of heterogeneity, activity/mechanism of action, and stability changes, as well as their impurity profiles, primarily resulting from their production.

In evaluating EV preparations according to Table 2, the three key aspects that are most crucial to CQA assessment and management are (i) their quantity and physical properties, (ii) their content and purity, and (iii) their potency/efficacy [105, 252]. For measuring EV quantity, the particle count or protein concentration is commonly used to establish dosing, although this is greatly affected by the purity of the EV preparation. Content and purity of EV preparations are typically assessed using proteomic/sequencing or targeted immunoblot/PCR approaches, which can detect both EV markers and co‐isolate markers. Potency is usually determined by in vitro assays.

**TABLE 2:** Generalized CQA Tests for EV Preparations.

CQA Category	Attribute/readout/methods
General Properties	Appearance (observations such as color and clarity)
	Osmolality
	pH
Identity	EV markers including tetraspanins (CD63, CD81, CD9, etc.) and EV‐associated markers (Alix, TSG101, syntenin‐1 etc.): ELISA, Western blot or antibody arrays
	Proteins, RNA or lipid signatures inherited from the source (e.g. MYADM, ADAM10, PS(36:1) for plasma, CD41/CD61, CD42b, CD62p for platelets)
	Particle size and concentration: Cryo‐electron microscopy (Cryo‐EM), atomic force microscopy (AFM), Nanoparticle tracking analysis (NTA), dynamic light scattering (DLS)
Purity	Morphology: Cryo‐EM (confirm bilayer membrane vesicles)
	Aggregation or fusion: DLS, NTA
	Total protein: Bicinchoninic acid assay, particle‐to‐protein ratio
	Total lipid content: Enrichment of phosphatidylserine (PS), lipid‐to‐particle ratio
	Purity by SE‐HPLC (size exclusion HPLC)
	Quantification of known co‐isolates (e.g. apolipoproteins, albumin, casein etc.)
	Zeta potential
	Subvisible particles: Micro‐flow imaging or light obscuration particles count
Impurities	Co‐isolated cellular contaminants, e.g., proteins from endoplasmic reticulum (Calnexin), Golgi (GM130), nucleus (Lamin B1), mitochondria (Prohibitin), and residual host cell DNA
Potency	Intended biological activity: (e.g. immunomodulation, angiogenesis, protective function, nucleic acid delivery efficacy etc.) measured by in vitro assays with defined dose ranges, timings and acceptance criteria
	Confirmation of specific active/desired cargo (RNAs, protein, lipids, metabolites) measured by PCR‐based methods, ELISA, mass spectrometry. These can be anchored to particle counts (i.e. miRNA copies per particle or protein concentration per particle)
	Target cell uptake efficiency and regulation of intended target (e.g. target gene suppressed by EV miRNA or activation of pathway by EV protein)
Safety	Immunogenicity risk
	Virus clearance validation
	Endotoxins/pyrogens
	Sterility (bacterial, fungal)
	Mycoplasma

For quantity, particle counts, or total protein are most often used to establish dosing. Yet neither metric is sufficient on its own: particle counts include NVEPs or insufficiently potent EVs, while co‐isolated proteins confound protein measurements. Historically, the particle‐to‐protein ratio was proposed as a purity benchmark [253]. While higher ratios can indicate less soluble protein contamination, such as albumin, the metric is misleading when particle‐rich but protein‐poor contaminants, like lipoproteins, are present, which artificially inflate the ratio [135]. As such, studies have reported particle: protein ratios from 10^6^–10^10^ particles/µg protein [24, 254, 255, 256]. Thus, this ratio can serve as a rough QC measure but should be interpreted alongside orthogonal methods such as atomic force microscopy (AFM) for stiffness profiling to distinguish vesicles vs lipoproteins, or cryogenic electron microscopy (cryo‐EM), to provide visual confirmation of bilayer vesicles [257, 258].

For purity and content, EV identity is typically demonstrated by the enrichment of positive markers such as tetraspanins and the absence of negative markers (e.g., GM130) [18]. Importantly, marker profiles are context‐specific. For instance, MYCT1, TSPAN14, MPIG6B, and MYADM were recently identified as better plasma EV markers, while CD41/CD61, CD42b, and CD62p better represent P‐EVs [144, 259]. Similarly, a recent paper describes proteomic and lipidomic signatures for human plasma EVs [22]. However, some co‐isolates may complex with EVs and contribute to the functional activity of an EV preparation, and should not be considered as contaminants [55, 260]. Thus, CQAs need to be established on a case‐by‐case basis, based on demonstrated efficacious preparations. Specific impurities, such as mycoplasma, endotoxins, and host cell DNA, can be detected using common commercially available assays. Specific unwanted or known detrimental cargo components can also be measured using ELISA, RT‐qPCR, and other assays. Together, the overall goal should be to obtain EV preparations with consistent compositions (of both EV and non‐EV markers) and functions.

For lipids, quantitative lipidomics by mass spectrometry provides information on both the identity of EVs and potential contaminants. EVs are typically enriched in bilayer lipids such as sphingomyelin (SM) and phosphatidylserine (PS). In contrast, detection of triacylglycerols (TG), cholesteryl esters (CE), or cardiolipin may indicate co‐isolated lipoproteins or organelle fragments. However, a recent perspective article highlights the technical challenges of carrying out robust EV lipidomic studies and the flaws in common lipid quantification methods (e.g., lipid type bias, isotope effect) [261]. As such, there is no consensus on an optimal particle‐to‐lipid ratio for EV benchmarking, since lipoproteins strongly confound the bulk lipid content. Instead, lipid class composition (e.g., enrichment of SM/PS vs presence of TG/CE compared to the originating cell) provides a more meaningful indicator of EV purity and origin. From an industrial manufacturing perspective, consistency remains the key, and a particle‐to‐lipid ratio can be established as an additional consistency benchmark, based on the EV origin (e.g., culture medium, plasma, platelets). A recent study by Rai and colleagues identified lipidomic signatures of circulating plasma EVs, identifying 52 lipids which were enriched specifically in EVs, with Phosphatidylserine PS(36:1) as a key marker [22].

The quantity of mRNA or miRNA (copies/EVs) can be used as a benchmark to validate the presence of known therapeutic nucleic acids. As mentioned in Section 1, studies commonly identify a particular miRNA as an “active” component of an EV preparation. Thus, to support these claims, the copy number and per‐particle and per‐protein ratios should be described [262]. Based on these, the administered dose of active nucleic acid can be approximated. In the published literature, there is a very wide range of RNA abundance, ranging from 0.000001 to > 1 copies per EV [262, 263, 264]. Again, this is likely attributable to both biological EV heterogeneity (the presence of rarer, miRNA‐rich vesicles) and methodological differences (isolation methods, purity (NVEPs), analytical methods etc.) between studies.

Finally, potency assays provide excellent functional CQAs. Standardized in vitro assays, such as fibroblast scratch migration (wound healing), endothelial tube formation (angiogenesis), macrophage polarization (immunomodulation), or cytoprotection under hypoxia/toxin exposure, can define dose‐response relationships and set potency thresholds [93]. These assays must be indication‐specific, performed with rigorous negative and positive controls, and reported quantitatively (e.g., % scratch closure or endothelial cell tube length at a defined time point and EV dose).

### 4.3 Characterization Challenges and Emerging Technologies

#### 4.3.1 Analytical Challenges

Despite the rapid technological advancements in recent years, EV characterization still faces several bottlenecks:

1. Purity‐yield tradeoffs in upstream sample preparation. There is currently no method that guarantees both high yield and high purity; UC co‐isolates NVEPs, SEC dilutes samples, and affinity‐based approaches are biased toward specific EV subpopulations. Emerging technologies (outlined in Section 3.3) are not yet in widespread research use, and each comes with its own trade‐offs yield and purity [265, 266].
2. Variability of results across analytical systems and between different laboratories. While guidelines such as MISEV aim to standardize methods and reporting, different research labs still report widely variable results from the same sample types. Traditional equipment, such as UC and NTA, can be operator‐dependent. In contrast, new methods, such as nFCM and interferometry, are more technically challenging to establish and have not been widely deployed [214, 267, 268].
3. Lack of calibration standards. EV reference standards such as synthetic particles and spiked EV‐mimics are emerging, but do not yet capture the biological heterogeneity of real samples. These standards can be used to validate EV isolation and purification, as well as to calibrate and standardize characterization techniques based on size, refractive index, or molecular markers [269, 270, 271].
4. Inherent high complexity of crude samples. While modern techniques such as nFCM and interferometry can more accurately measure EV samples <100 nm, crude, unprocessed samples contain abundant NVEPs, which confound particle counts and size distributions. Thus, no tool can truly report EV recovery, since current instruments can only obtain reliable measurements after purification. Therefore, these analytics do not account for EVs that are lost during the purification process [272, 273, 274].
5. Throughput, cost, and technical requirements. The new processes for isolation and characterization, outlined earlier, are not yet commonplace in basic research labs. Many of the emerging techniques mentioned earlier (e.g., AF4, EVOs, DLD, etc.) require a greater degree of technical expertise to set up, validate, and use successfully than UC, SEC, precipitation, or filtration approaches [275].

#### 4.3.2 Emerging Solutions

To reduce variability across laboratories, the community is encouraged to move towards orthogonal, multi‐assay strategies, ideally anchored by reference standards and regulatory‐style QC frameworks. The appreciation of EV heterogeneity is driving interest in calibration materials and universal benchmarks that can improve the comparability of results across instruments and sample types [276]. The complexity of crude samples is steering innovation toward label‐free, high‐specificity tools that can differentiate vesicles from proteins, lipoproteins, and debris, ideally in minimally processed fluids [277]. Many new products, such as the Formulatrix µPulse or Sartoflow Smart (benchtop TFF), Apogee MicroPlus (nFCM), NanoView ExoView (interferometry), Izon Exoid (tunable resistive pulse sensing, TRPS), and Waters Eclipse (AF4), now offer more user‐friendly devices for advanced analytical techniques. These may become more widely used in the future, in the way that DLS, NTA, and cryo‐EM are commonplace today. There is also a growing emphasis on integrated platforms that combine isolation with characterization in a single workflow, reducing sample loss and enabling closer tracking of recovery [275, 278]. For example, a multi‐stage enrichment workflow described in HEK293F‐derived EVs demonstrated how QC‐driven processing and characterization can ensure consistency and higher confidence in downstream analyses [279].

In December 2025, the National Institute of Standards and Technology (NIST) published an inter‐method characterization study, as a first step towards producing standardized reference materials [280]. The study compared cryoEM, particle tracking, AF4 and microfluidic RPS to measure the concentration and size of EVs isolated from hTERT MSCs and LNCaP cells by TFF. The authors found that calculated particle concentrations varied by up to two orders of magnitude (10^10^–10^12^ particles/mL), across different measurement methods. Similarly, small RNA sequencing of LNCaP‐EV cargo was performed at two independent labs, which reported 694 miRNAs and 740 miRNAs, respectively, with 485 miRNAs being detected in both labs, albeit in different relative abundances. This again illustrates that reported EV properties can vary significantly between measurement platforms and different labs. As such, claims of EV composition and function should be framed at the level of a defined EV preparation, anchored to its source, isolation, and characterization workflow, and assay context, rather than treated as inherent properties of the broad class of EVs.

## 5 Future Outlook

### 5.1 Translational Outlook

As outlined in Section 1, many promising claims have been made regarding EVs as therapeutics, including intrinsic biocompatibility, low immunogenicity, natural tropism, highly efficient nucleic acid delivery, and many others. Thousands of research papers have demonstrated the roles of EVs in paracrine signaling and have shown therapeutic effects of EV preparations from different sources across multiple disease models. It is also clear that EV preparations contain complex mixtures of regulatory molecules that can exert strong biological effects via a wide variety of mechanisms. Yet, the practical clinical use of EVs as therapeutics has not yet been realized, aside from a small number of early‐stage clinical trials [105, 281, 282, 283].

Regarding clinical‐scale EV production, recent work has demonstrated how a GMP‐style, continuous production platform can be implemented in practice. A SuperPro‐modeled simulation illustrated that large‐scale MSC‐EV production is both feasible and economically viable, using 3D microcarrier perfusion for cell expansion followed by a combination of TFF, nuclease treatment, and anion‐exchange chromatography to purify EVs [284]. Their model projected production of ∼239 EV doses (1 × 10^1^ ^1^ particles) per batch, at a cost of €1700 to €3100 per dose. As a practical follow‐on to the simulation, Ulpiano et al. ran the platform end‐to‐end under xeno‐free conditions, achieving MSC‐EV yields of ∼1.3 × 10^4^ EVs/cell/day, with high purity (∼5.5 × 10^9^ particles/µg) [127]. These data demonstrate that continuous, GMP‐style EV manufacturing at meaningful clinical scales is feasible with current technology.

Currently, several commercialization efforts and larger clinical trials are ongoing, including Direct Biologics/ExoFlo (MSC‐EVs for acute respiratory distress syndrome (NCT04493242), Crohn's Disease, and ulcerative colitis), Rion LLC (P‐EVs for diabetic ulcers, osteoarthritis, aesthetics, myocardial infarction), and PranaX (BM‐MSC‐EV with siRNA targeting KRAS G12D for pancreatic ductal adenocarcinoma) [185]. Other academic trials include SECRET‐HF (NCT05774509), which assess the safety of iPSC‐derived cardiac progenitor EVs in patients with cardiomyopathy. However, it is notable that several previously prominent EV clinical programs have been discontinued or suspended since 2023. Codiak BioSciences, which developed several engineered EV products, filed for bankruptcy in March 2023. Similarly, ExoPharm discontinued development of Plexaris and Cevaris, pivoting the company away from EV therapeutics entirely. Other IND‐cleared programs, such as Aruna Bio's AB126 for acute ischemic stroke, have not yet commenced patient dosing due to funding constraints. Thus, as of early 2026, only a handful of dedicated EV therapeutics are actively advancing through clinical development. High attrition rates are typical for early‐stage biotech (e.g. cell therapy, gene therapy, etc.) and are not exclusive to EV therapies. However, this nevertheless highlights the significant translational gap between preclinical promise and the scientific, manufacturing, regulatory, and financial hurdles discussed throughout this review. As such, the results of these trials may serve to further fuel, or perhaps quell, the hype surrounding EV‐based therapeutics.

### 5.2 Future Directions

In the future, artificial intelligence and machine‐learning algorithms are likely to transform single‐EV analysis by automating image processing, classifying vesicles from multimodal datasets (e.g., Raman spectra, AFM, nFCM), and identifying hidden correlations between EV phenotype and function, thereby accelerating biomarker and therapeutic discovery [276].

While the direct translation of natural EVs is clearly challenging, insights from EV research are already inspiring the development of EV mimetics, in which synthetic lab‐made vesicles can be produced with features similar to those of natural EVs. For example, Staufer and colleagues produced synthetic vesicles from a lipid composition found in natural EVs, and showed that decorating the membrane with CD9, CD63, and CD81 improved uptake into cells. They were also able to load a precise dose of miRNA (54 ± 5.6 miRNA molecules/vesicle), enabling a greater degree of control compared to EV preparations from natural sources [285]. Synthetic EVs, in principle, could recapitulate the benefits of natural EVs while enabling scalable and more consistent mass production, with more precise dosing based on defined active components [286]. Using such systems, synthetic EV mimetics could be developed to carry optimal cargo and optimal targeting moieties for a specific indication, while avoiding unwanted cargo [38, 287, 288]. The use of proprietary fabrication technology and novel targeting/fabrication formulations will also allow intellectual property protection and commercialization. For example, recent successes with lipid nanoparticle mRNA vaccines (Comirnaty, SpikeVax), siRNAs (Patisiran), and a longer history of small molecules (e.g. liposomal irinotecan (Onivyde), doxorubicin (LipoDox/Caelyx)) and others demonstrate the feasibility of translating synthetic vesicles to clinical use.

EVs in medicine exist at an intersection of hype and unrealized potential. Their unique biology and broad therapeutic potential have fueled an exponential rise in research publications, direct‐to‐consumer products, and clinical trials [289, 290]. Enthusiasm, particularly in commercial applications, has outpaced regulation, resulting in market offerings that risk undermining confidence in the field. As we have highlighted, some of the most appealing claims about EVs (e.g. selective targeting, barrier traversal, efficacy driven by defined cargo molecules) have been challenged by newer measurement techniques. Several concrete milestones will likely determine whether EV therapeutics progress from early‐phase candidates to approved medicines. Positive results from Phase 3 trials with transparent CQA reporting, such as the ongoing EXTINGUISH‐ARDS and planned RION DFU studies, would provide the first definitive clinical validation. In parallel, the establishment of reference standards and greater regulatory convergence across jurisdictions would substantially lower the barrier for developers pursuing multinational programs. Resolving questions of EV doses (particle, protein, specific cargo, etc.) will also be critical, and it will likely need to be addressed empirically for each indication rather than through consensus on a single universal metric.

Lastly, rapid advances in EV isolation strategies, single‐particle analytics, and continuous biomanufacturing are beginning to provide the tools needed to standardize preparations and align them with regulatory expectations for biologics. These techniques will enable us to recognize the entire makeup of an EV preparation (e.g., a heterogeneous mix of vesicle subpopulations, co‐isolated proteins, lipoproteins, and metabolites) as the true functional unit while effectively characterizing and standardizing those components. In this respect, clear reporting following MISEV guidelines will greatly enhance the impact of work in this field. Through collaboration across academia, industry, and regulators, we hope that EVs will succeed as a legitimate new class of biologic medicines for cancers, cardiovascular diseases, neurodegenerative disorders, and more.

## Conflicts of Interest

The authors declare no conflict of interest.

## Data Availability

The authors have nothing to report.
